# Inactivation of nuclear GSK3β by Ser^389^ phosphorylation promotes lymphocyte fitness during DNA double-strand break response

**DOI:** 10.1038/ncomms10553

**Published:** 2016-01-29

**Authors:** Tina M. Thornton, Pilar Delgado, Liang Chen, Beatriz Salas, Dimitry Krementsov, Miriam Fernandez, Santiago Vernia, Roger J. Davis, Ruth Heimann, Cory Teuscher, Michael S. Krangel, Almudena R. Ramiro, Mercedes Rincón

**Affiliations:** 1 Department of Medicine/Immunobiology, University of Vermont, Burlington, Vermont 05405, USA; 2 B Cell Biology Lab, Centro Nacional de Investigaciones Cardiovasculares (CNIC), Madrid 328029, Spain; 3 Department of Immunology, Duke University Medical Center, Durham, North Carolina 27710, USA; 4 Program in Molecular Medicine, University of Massachusetts, Worcester, Massachusetts 01605, USA; 5 Howard Hughes Medical Institute, Worcester, Massachusetts 01605, USA; 6 Department of Medicine/Radiology, University of Vermont, Burlington, Vermont 05405, USA

## Abstract

Variable, diversity and joining (V(D)J) recombination and immunoglobulin class switch recombination (CSR) are key processes in adaptive immune responses that naturally generate DNA double-strand breaks (DSBs) and trigger a DNA repair response. It is unclear whether this response is associated with distinct survival signals that protect T and B cells. Glycogen synthase kinase 3β (GSK3β) is a constitutively active kinase known to promote cell death. Here we show that phosphorylation of GSK3β on Ser^389^ by p38 MAPK (mitogen-activated protein kinase) is induced selectively by DSBs through ATM (ataxia telangiectasia mutated) as a unique mechanism to attenuate the activity of nuclear GSK3β and promote survival of cells undergoing DSBs. Inability to inactivate GSK3β through Ser^389^ phosphorylation in Ser^389^Ala knockin mice causes a decrease in the fitness of cells undergoing V(D)J recombination and CSR. Preselection-*Tcrβ* repertoire is impaired and antigen-specific IgG antibody responses following immunization are blunted in Ser^389^GSK3β knockin mice. Thus, GSK3β emerges as an important modulator of the adaptive immune response.

## Abstract (web summary)

Double stranded DNA breaks are generated during rearrangements of lymphocyte antigen receptors. Here the authors show that the DNA breaks induce phosphorylation of nuclear GSK3β at Ser^389^/Thr^390^, protecting the activated lymphocytes from necroptosis-mediated cell death.

## Graphical abstract

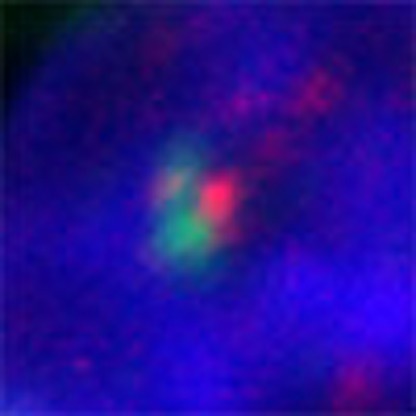

Glycogen synthase kinase 3β (GSK3β) is a serine threonine protein kinase abundantly expressed in all cells and tissues^1^. GSK3β is present predominantly in the cytoplasm, but also within the nucleus in response to pro-apoptotic stimuli, although the function of nuclear GSK3β is unclear^2^,^3^. GSK3β plays a critical role in determining the balance between cell survival and death^4^. Deletion of GSK3β results in lethality during embryonic development^5^. Unlike most kinases, GSK3β is constitutively active and high levels of GSK3β activity are associated with its role in promoting cell death^4^. To maintain cell survival, active mechanisms are required to restrain GSK3β activity^6^,^7^,^8^. Although cell death also plays an important role during T- and B-cell development and the immune response, little is known about the contribution of GSK3β to adaptive immune responses. Pharmacological inhibitors that inhibit the activity of both GSK3β and its closely related kinase GSK3α, have been shown to interfere with thymocyte development at the double negative (DN)3 stage *in vitro*^9^. Pharmacological inhibition of GSK3β/GSK3α activity can reduce activation-induced cell death of CD4 cells^10^. In contrast, other studies have proposed that GSK3α is primarily required for Th1 differentiation while GSK3β is primarily required for Th17 differentiation^11^. It remains unknown whether GSK3β plays a role in B-cell development or activation.

The best-characterized mechanism to repress GSK3β is the phosphorylation of Ser^9^ primarily by Akt (refs 12, 13). The flexible N terminus of GSK3β containing phospho-Ser^9^ acts as an intrinsic competitive inhibitor of this kinase by folding into the active site and competing with substrate binding^14^. High levels of phospho-Ser^9^ GSK3β can be detected without external stimulation in most cells of the immune system. However, unexpectedly, GSK3β Ser^9^Ala knockin (KI) mice do not have a defect in cell survival within the immune system or other tissues^15^. This could be due to the compensatory effect by GSK3α because increased activation-induced cell death of CD4 cells was found in double mutant mice for GSK3β Ser^9^Ala and GSK3α Ser^21^Ala (analogue to Ser^9^ in GSK3β)^16^. Increased GSK3 activity in the Ser^9^Ala GSK3β/Ser^21^Ala GSK3α double mutant mice promoted polarization of CD4 T cells to Th17 (ref. 17). No studies, to the best of our knowledge, have reported the function of GSK3β Ser^9^ inactivation in B cells.

We have identified that GSK3β can also be inactivated by phosphorylation at Thr^390^(human)/Ser^389^ (mouse) by p38 mitogen-activated protein kinase (MAPK)^18^. Phosphorylation of GSK3β at Ser^389^/Thr^390^ inactivates GSK3β to a similar degree as Ser^9^ phosphorylation by Akt (ref. 18). While Akt, inhibits both GSK3α and GSK3β, p38 MAPK does not phosphorylate GSK3α (ref. 18). Interestingly, phosphorylation of Ser^9^ is ubiquitously present under normal conditions, Ser^389^ phosphorylation is restricted to the thymus, spleen and brain^18^. Thus, Ser^389^ phosphorylation represents an alternative pathway to restrain GSK3β activity, but it remains unclear which stimuli require this pathway and its function in cell death/survival. Here we show that phosphorylation of GSK3β at Ser^389^/Thr^390^ is specifically induced in response to DNA double-strand breaks (DSBs) and targets nuclear GSK3β. We show that DSBs naturally generated during V(D)J recombination in T- and B-cell receptors, and during class switch recombination (CSR) of the immunoglobulin genes in activated B cells^19^, inactivate nuclear GSK3β through Ser^389^ phosphorylation. We demonstrate that this pathway plays an important role in the fitness of T and B cells during DSB repair.

## Results

### Phosphorylation of GSK3β on Ser^389^ is induced by DSB

**Figure 1:**
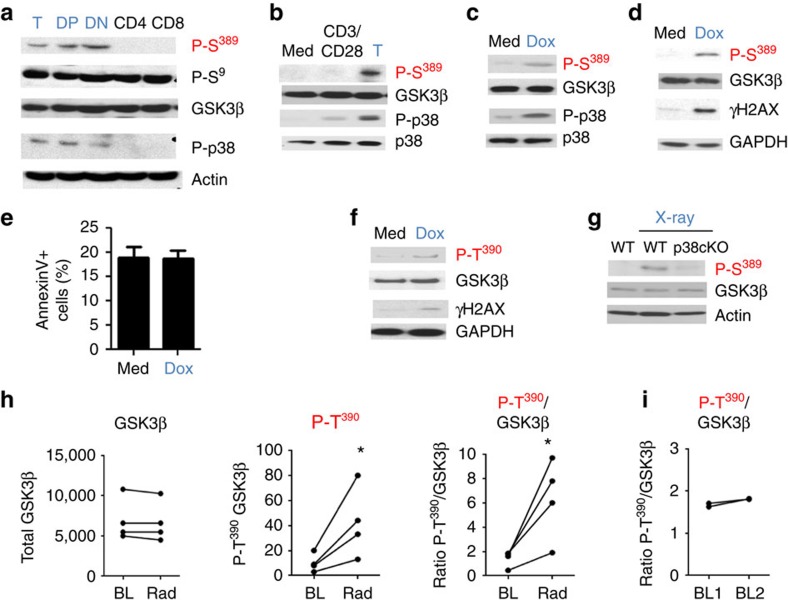
Inactivation of GSK3β by p38 MAPK is specifically induced by DSBs. (**a**) Western blot analysis of P-S^389^ GSK3β, P-S^9^ GSK3β, GSK3β, P-p38 MAPK and p38 MAPK in total thymocytes (T), double positive thymocytes (DP), double negative thymocytes (DN), mature CD4 T cells and mature CD8 T cells. Actin is shown as a loading control. (**b**) Mouse CD4 splenocytes were treated with media alone (Med) or stimulated with anti-CD3 and anti-CD28 Abs for 18 h and the levels of P-S^389^ GSK3β, GSK3β, P-p38 MAPK and p38 MAPK were determined by western blotting. Thymocytes are included as a positive control. (**c**) Mouse CD4 splenocytes were treated with media alone (Med) or doxorubicin (Dox) for 18 h and the levels of P-S^389^ GSK3β, total GSK3β, P-p38 MAPK and total p38 MAPK were determined by western blotting. (**d**) Mouse CD4 splenocytes were treated with media alone (Med) or doxorubicin (Dox) for 18 h. Levels of P-S^389^ GSK3β, total GSK3β and γH2AX were determined by western blotting. GAPDH is shown as a loading control. (**e**) Mouse CD4 splenocytes were treated with doxorubicin for 18 h and cell death was measured by AnnexinV staining and flow cytometry (*n*=3, ±s.e.m.). (**f**) Human CD4 cells were treated with media alone (Med) or doxorubicin (Dox) for 18 h. P-T^390^ GSK3β, total GSK3β and γH2AX were examined by western blotting. (**g**) WT and T-cell-specific p38α conditional knockout (cKO) mice were left unexposed or exposed to 4 Gy of X-rays. After 1.5 h, CD4 splenocytes were isolated and P-S^389^ GSK3β and total GSK3β assessed by western blotting. Actin is shown as a loading control. (**h**) Relative levels of total GSK3β, P-T^390^ GSK3β and the relative ratio of P-T^390^ GSK3β to total GSK3β at baseline (BL) and after radiotherapy (Rad) in CD4 cells from breast cancer patients, determined by the Odyssey system (*n*=4). (**i**) Relative ratio of P-T^390^ GSK3β to total GSK3β in CD4 cells from healthy donors isolated in two different days (baseline 1 and 2) for each subject (*n*=3). *P value<0.05 as determined by paired *t*-test. (**a**–**g**) Data are representative of three or more independent experiments.

While Ser^9^ phosphorylation of GSK3β is ubiquitous, phosphorylation of GSK3β Ser^389^ occurs predominately in the thymus^18^. To determine whether phosphorylation of Ser^389^ on GSK3β was regulated during T-cell development, we examined phospho-Ser^389^ GSK3β in DN and double positive (DP) thymocytes as well as mature CD4 and CD8 cells. Interestingly, while total GSK3β and phospho-Ser^9^ GSK3β did not change during development, high levels of phospho-Ser^389^ GSK3β were detected in both DN and DP thymocytes, but not in mature CD4 and CD8 cells (Fig. 1a). Consistent with our previous results^18^, phosphorylation of GSK3β on Ser^389^ correlates with the presence of active p38 MAPK (Fig. 1a).

Unlike peripheral naive T cells, both DN and DP thymocytes are rapidly proliferating. To determine whether proliferation promoted Ser^389^ phosphorylation of GSK3β, we activated CD4 cells with anti-CD3 and anti-CD28 antibodies (Abs). Although p38 MAPK was activated upon antigen stimulation, no phospho-Ser^389^ GSK3β could be detected in activated CD4 cells indicating that proliferation does not induce phospho-Ser^389^ GSK3β (Fig. 1b). We have shown that p38 MAPK is activated by V(D)J-mediated DSBs in thymocytes^20^. To investigate whether phospho-Ser^389^ GSK3β was also triggered by DSBs, we examined Ser^389^ phosphorylation of GSK3β in CD4 cells following treatment with doxorubicin, a chemotherapeutic agent known to generate DSBs^21^. Interestingly, doxorubicin elicited the phosphorylation of GSK3β on Ser^389^ and this correlated with the activation of p38 MAPK (Fig. 1c) and the induction of DSBs as determined by the presence of γH2AX (Fig. 1d). The low dose of doxorubicin that we used to induce DSBs did not cause significant death in CD4 cells (Fig. 1e). Similar to mouse CD4 cells, phospho-Thr^390^ was induced by doxorubicin in human CD4 cells isolated from healthy controls (Fig. 1f).

To examine induction of phospho-Ser^389^ GSK3β *in vivo* by DSBs, mice were irradiated and CD4 cells were purified from spleen after exposure. X-irradiation induced Ser^389^ phosphorylation of GSK3β in CD4 cells (Fig. 1g). Ser^389^ phosphorylation was p38α MAPK dependent, since only marginal levels of phospho-Ser^389^ GSK3β could be detected in CD4 cells from T-cell conditional p38α MAPK knockout (p38c KO) mice (Fig. 1g). To determine whether *in vivo* exposure to radiation could induce Thr^390^ phosphorylation of GSK3β in humans, we performed a pilot study with breast cancer patients undergoing local radiotherapy as the first regimen of therapy. CD4 cells were isolated from peripheral blood collected before beginning the treatment (base line). Patients received a daily dose of radiotherapy for four consecutive days and CD4 cells were isolated from blood collected 24 h after the last dose. While total GSK3β levels remained unchanged by the treatment (Fig. 1h), following radiotherapy phospho-Thr^390^ GSK3β was increased over baseline in all four patients (Fig. 1h). We also examined phospho-Thr^390^ GSK3β levels at two different time points (4–6 days apart) in CD4 cells from healthy untreated volunteers, and no changes over time were detected (Fig. 1i). Thus, phosphorylation on Ser^389^/Thr^390^ regulates GSK3β selectively in response to DSBs in both mouse and human.

### V(D)J induces phospho-Ser^389^ GSK3β in the nucleus

**Figure 2:**
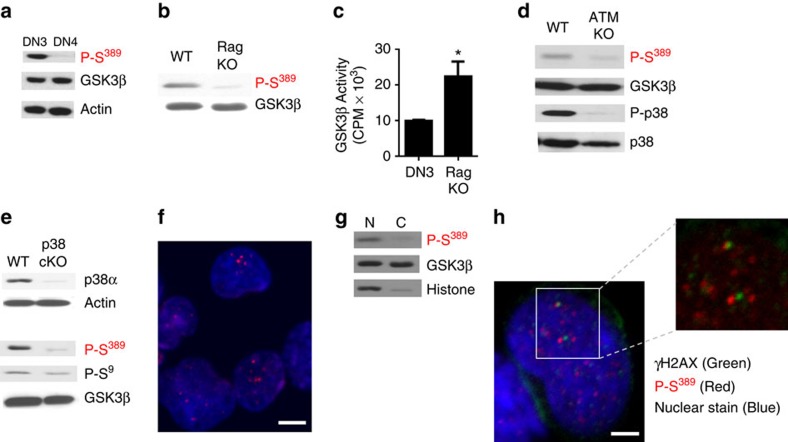
DSBs generated by V(D)J in developing T cells induce inactivation of GSK3β by p38 MAPK. (**a**) DN3 and DN4 thymocytes were examined for P-S^389^ GSK3β and total GSK3β by western blot analysis. Actin is shown as a loading control. (**b**) The levels of P-S^389^ GSK3β and total GSK3β in DN3 thymocytes from WT and Rag KO were examined by western blot analysis. (**c**) GSK3β activity in lysates from DN3 thymocytes from WT and Rag KO mice were determined using *in vitro* kinase activity assays (*n*=3,±s.e.m.). *P<0.05 as determined by *t*-test. (**d**) The levels of P-p38 MAPK, total p38 MAPK, P-S^389^ GSK3β and total GSK3β in DN3 thymocytes from WT and ATM KO mice were determined by western blot analysis. (**e**) DN3 thymocytes from WT and p38α conditional KO (p38cKO) mice were examined for p38α, P-S^389^ GSK3β, P-S^9^ GSK3β and total GSK3β by western blot analysis. Actin is shown as a loading control. (**f**) DN3 thymocytes were examined by immunostaining and confocal microscopy for the presence of P-S^389^ GSK3β (red) and TOPRO nuclear stain (blue). Scale bar, 5 μm. (**g**) Western blot analysis for P-S^389^ GSK3β and total GSK3β using nuclear and cytosolic extracts from DN thymocytes. Histone is shown as a marker for the nuclear fraction. (**h**) DN3 thymocytes were examined by immunostaining and confocal microscopy for the presence of γH2AX (green), P-S^389^ GSK3β (red) and TOPRO nuclear stain (blue). Scale bar, 2 μm. Data are representative of three or more independent experiments.

DSBs are also naturally produced in lymphocytes during V(D)J recombination to generate the coding T-cell and B-cell receptor genes^22^,^23^,^24^. V(D)J-mediated DSBs also trigger DNA damage and repair responses^23^. At the DN3 stage of development, thymocytes undergo V(D)J recombination of the TCRβ locus to generate a functional TCRβ that provides a signal to terminate recombination and promote differentiation to the DN4 stage. Although the levels of total GSK3β were comparable between DN3 and DN4 thymocytes, high levels of phospho-Ser^389^ were only detected in DN3 thymocytes (Fig. 2a). To show that phospho-Ser^389^ GSK3β was dependent on V(D)J recombination, we examined DN3 thymocytes from wild-type (WT) and recombination activating gene (RAG)-deficient mice that cannot undergo V(D)J recombination due to the lack of RAG recombinase^25^. Phospho-Ser^389^ GSK3β was much more abundant in WT DN3 thymocytes than in RAG KO thymocytes (Fig. 2b). To determine whether the increased level of phospho-Ser^389^ GSK3β correlated with lower GSK3β activity, *in vitro* kinase assays were performed. Lower GSK3β activity was present in WT thymocytes than in RAG KO thymocytes (Fig. 2c). Ataxia telangiectasia mutated (ATM) is a kinase activated by DSBs including V(D)J-mediated DSBs and it is a major player in the DSB-repair response^26^. To address whether phosphorylation of GSK3β on Ser^389^ in DN3 thymocytes was triggered by DSBs or the DSB-repair response we examined DN3 thymocytes in ATM KO mice. Phospho-Ser^389^ GSK3β was diminished in DN3 thymocytes from ATM KO mice (Fig. 2d). Consistent with previous studies *in vitro* DSBs^27^, activated p38 MAPK was almost absent in ATM KO thymocytes (Fig. 2d). In addition, analysis of phospho-Ser^389^ in DN3 thymocytes from T cell-conditional p38α MAPK KO mice showed minimal levels compared to WT DN3 thymocytes (Fig. 2e). In contrast, phospho-Ser^9^ GSK3β levels were not affected (Fig. 2e). Thus, the restricted presence of GSK3β phosphorylated on Ser^389^ in thymocytes *in vivo* is due to the presence of V(D)J-induced DSB-repair response as a signalling pathway to inactivate GSK3β through ATM and p38 MAPK.

We have previously shown that DSBs result in the selective accumulation of p38 MAPK in the nucleus^28^. Phospho-Ser^389^ GSK3β localized selectively to the nucleus as shown by immunostaining and confocal analysis in WT DN3 thymocytes (Fig. 2f) and this was confirmed by western blot analysis (Fig. 2g). A similar nuclear distribution was found for human phospho-Thr^390^ GSK3β in a human cancer cell line following treatment with doxorubicin (Supplementary Fig. 1a), and this was prevented by inhibiting ATM (Supplementary Fig. 1b). The focal distribution of phospho-Ser^389^ in the nucleus of DN3 thymocytes was reminiscent of γH2AX foci at the DSBs generated by V(D)J recombination at the TCRβ locus. To determine whether phospho-Ser^389^ GSK3β was recruited to the DSBs, we performed co-immunostaining for both γH2AX and phospho-Ser^389^ in DN3 thymocytes. Interestingly, the most prominent phospho-Ser^389^ foci were adjacent to the γH2AX foci (Fig. 2h). Thus, phosphorylation of GSK3β on Ser^389^ is induced by V(D)J-mediated DSBs in thymocytes to preferentially inactivate nuclear GSK3β.

### Phospho-Ser^389^ GSK3β in B cells undergoing CSR-DSBs

**Figure 3:**
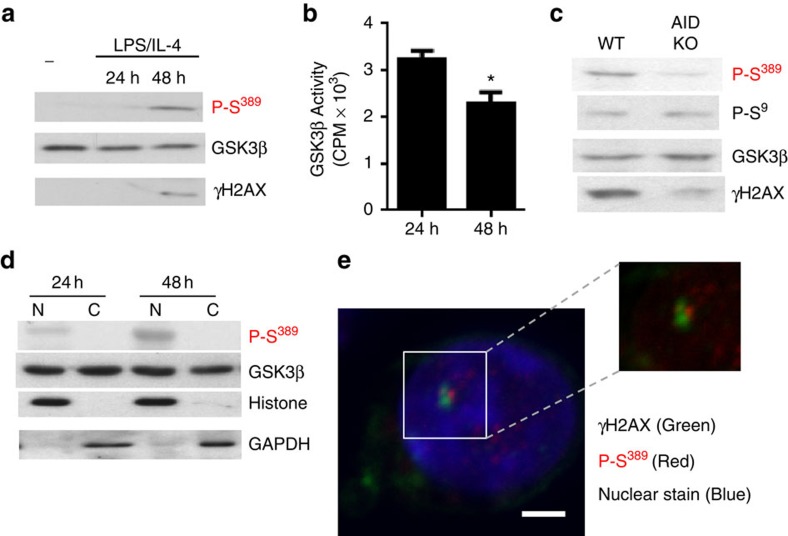
DSBs generated by antibody class switch recombination induce inactivation of GSK3β by p38 MAPK. (**a**) Fresh B cells (−) and B cells activated with LPS and IL-4 for 24 and 48 h were examined by western blotting for P-S^389^ GSK3β, total GSK3β and γH2AX. (**b**) GSK3β activity in B cells activated with LPS and IL-4 for 24 and 48 h were examined by *in vitro* kinase assays (*n*=3, ±s.e.m.). *P<0.05 as determined by *t*-test. (**c**) B cells from WT and AID KO mice were stimulated for 48 h and the levels of P-S^389^ GSK3β, P-S^9^ GSK3β, total GSK3β and γH2AX were determined by western blot analysis. (**d**) Western blot analysis for P-S^389^ GSK3β and total GSK3β using nuclear and cytosolic extracts from B cells activated for 24 and 48 h. GAPDH and histone are shown as controls. (**e**) B cells activated with LPS and IL-4 for 48 h were examined by immunostaining and confocal microscopy for the presence of γH2AX (green), P-S^389^ GSK3β (red) and TOPRO nuclear stain (blue). Scale bar, 3 μm. Data are representative of three or more independent experiments.

CSR of the Ig heavy chain locus in B cells initiated by activation-induced cytidine deaminase (AID) also leads to the generation of DSBs and involves components of the DNA repair machinery^22^,^24^,^29^. B cells were isolated from the spleen and activated with lipopolysaccharide (LPS) and interleukin-4 (IL-4) to induce CSR for the production of IgG1. Phospho-Ser^389^ GSK3β was induced following activation and correlated with the presence of DSBs as determined by γH2AX (Fig. 3a). Analysis of GSK3β activity in whole-cell lysates from activated B cells showed lower activity at 48 h of activation correlating with the induction of GSK3β Ser^389^ phosphorylation (Fig. 3b). To demonstrate that AID-mediated DSBs were required to induce phosphorylation of Ser^389^, we examined B cells from WT and AID KO mice following stimulation. Correlating with a significant reduction in DSBs as determined by γH2AX (Fig. 3c), phospho-Ser^389^ GSK3β was reduced in the AID KO B cells compared with WT B cells (Fig. 3c). In contrast, levels of phospho-Ser^9^ GSK3β were not affected in AID KO B cells (Fig. 3c). We investigated the subcellular distribution of phospho-Ser^389^ GSK3β in B cells undergoing CSR. Phospho-Ser^389^ GSK3β was predominately in the nucleus of activated B cells (Fig. 3d) while total GSK3β was present in both the nucleus and cytosol (Fig. 3d). We also examined whether phospho-Ser^389^ GSK3β was associated with CSR-generated DSBs by co-immunostaining of γH2AX and phospho-Ser^389^ GSK3β. Phospho-Ser^389^ foci were adjacent to the γH2AX foci in activated B cells (Fig. 3e). Thus, DSBs generated in B cells undergoing CSR selectively cause the inactivation of nuclear GSK3β through phosphorylation on Ser^389^.

### TCRβ rearrangement is impaired in GSK3β Ser^389^ KI mice

**Figure 4:**
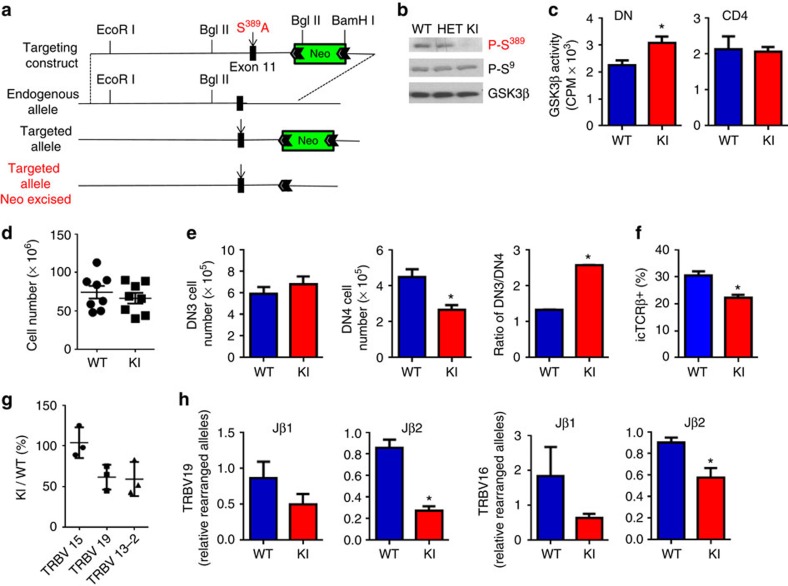
Survival of thymocytes undergoing TCRβ rearrangement at the DN3 stage of development is dependent of inactivation GSK3β by phosphorylation on Ser^389^. (**a**) Diagram depicting the GSK3β Ser^389^Ala knockin (KI) construct, the endogenous GSK3β allele containing exon 11 (black box), the targeted allele with the neomycin selection cassette still present and the targeted allele with the neomycin cassette removed by Cre recombinase. White notched arrow heads represent LoxP sites. (**b**) Whole-cell lysates from thymocytes of WT, heterozygous (Het) and homozygous GSK3β-KI mice were analysed by western blot analysis for P-Ser^389^ GSK3β, P-Ser^9^ GSK3β and total GSK3β. (**c**) GSK3β activity in DN thymocytes and mature CD4 splenocyte from WT and GSK3β-KI mice were examined by *in vitro* kinase assays (*n*=3). (**d**) Total number of thymocytes in WT (circles) and GSK3β-KI mice (squares) (*n*=8). (**e**) Total numbers of DN3 and DN4 thymocytes and the ratio of DN3/DN4 thymocytes from WT and GSK3β-KI mice (*n*=6). (**f**) Intracellular staining of TCRβ and flow cytometry analysis of DN3 thymocytes from WT and GSK3β-KI mice (*n*=4). The percentage of iTCRβ positive cells within the DN3 population is shown. (**g**) The percent fluorescence signal intensity of KI Vβ CDR3 region peaks relative to WT Vβ CDR3 region peaks (*n*=3 independent mice). (**h**) Real-time PCR was used to examine recombination between Vβ (TRBV19 and TRVB16) and Jβ (Jβ1.1 or Jβ2.1) in genomic DNA from DN thymocytes. Signals were normalized to the non-recombining gene. (*n*=3 independent mice for each genotype). (**c**,**e**,**f**,**h**) Data are shown as mean±s.e.m. *P<0.05 as determined by *t*-test. Data are representative of three or more independent experiments.

To determine the function of the phosphorylation of GSK3β at Ser^389^/Thr^390^ relative to classical Ser^9^ phosphorylation, we generated GSK3β-KI mice where Ser^389^ of GSK3β was replaced with Ala to prevent the C-terminal phosphorylation of GSK3β (Fig. 4a). Western blot analysis confirmed the absence of phospho-Ser^389^ GSK3β in thymocytes from homozygous GSK3β-KI mice, while normal levels were present in heterozygous GSK3β-KI mice (Fig. 4b). Ser^9^ phosphorylation of GSK3β was not affected by the Ser^389^Ala mutation (Fig. 4b), showing that these two residues represent independent pathways to regulate GSK3β activity. To address the relative contribution of Ser^389^ phosphorylation to the overall GSK3β activity, we assayed GSK3β activity in DN thymocytes and mature CD4 cells from WT and GSK3β-KI mice. An increase in GSK3β activity was detected in DN thymocytes from GSK3β-KI mice but no difference was detected in mature CD4 cells correlating with the absence of phospho-Ser^389^ GSK3β in mature CD4 cells (Fig. 4c). Thus, phosphorylation of Ser^389^ contributes to restraining the activity of GSK3β, independent of Ser^9^ phosphorylation.

S^389^A-GSK3β-KI mice were fertile and no developmental abnormalities or gross alterations were detected under physiological conditions. The percentage and number of mature CD4 and CD8 cells in the spleen and lymph nodes was not altered in GSK3β-KI mice (Supplementary Fig. 2a–c). The fact that no phospho-Ser^389^ GSK3β could be found in activated CD4 cells (Fig. 1b) suggested that this pathway is not essential for activation of CD4 cells. Analysis of proliferation of activated CD4 cells from WT and GSK3β-KI mice showed no obvious difference (Supplementary Fig. 3a). The number of cells recovered upon activation was also comparable (Supplementary Fig. 3b) and there was no difference in IL-2 production (Supplementary Fig. 3c). Thus, correlating with the selective inactivation of GSK3β through Ser^389^ phosphorylation in response to DSBs, this pathway does not seem to be required for normal activation of CD4 cells.

We next assessed the contribution of this pathway to thymocyte development. No difference in the total number of thymocytes (Fig. 4d), or in the numbers and percentage of DP and single positive (SP) thymocytes (Supplementary Fig. 4a,b), was detected between WT and S^389^A-GSK3β-KI mice. We examined Vα to Jα rearrangements in DP thymocytes to determine if inability to inactivate GSK3β during Vα–Jα recombination could impair the TCRα repertoire. Analysis of Vα to Jα rearrangements using genomic DNA from isolated total DP thymocytes suggested that GSK3β Ser^389^Ala mutation had no effect in survival of DP thymocytes during TCRα recombination (Supplementary Fig. 5). The number of total and percentage of DN thymocytes in GSK3β-KI mice was not significantly altered (Supplementary Fig. 4a,b). However, examination of early thymocyte development within the DN population showed a reduction in the percentage and total number of DN4 thymocytes in GSK3β-KI mice (Fig. 4e; Supplementary Fig. 4a). The reduction of DN4 thymocyte number was associated with an increase in the DN3/DN4 ratio (Fig. 4e). We examined whether preventing inactivation of GSK3β by Ser^389^ phosphorylation during TCRβ-V(D)J recombination affected the number of DN3 thymocytes that successfully survive β-rearrangement. Intracellular staining for TCRβ in DN3 thymocytes showed a lower frequency of DN3 thymocytes with successfully rearranged TCRβ in GSK3β-KI mice (Fig. 4f). Thus, inactivation of GSK3β through Ser^389^ phosphorylation contributes to the fitness of the DN3 thymocyte pool undergoing V(D)J recombination.

To determine whether this could result in an altered distribution of TCRβ specificities in mature T cells, CD4 cells freshly isolated from the spleen of WT and GSK3β-KI mice were used for TCRβ spectrotyping analysis (Supplementary Fig. 6). We detected a lower frequency of TRBV19 and TRBV13-2 in CD4 cells from GSK3β-KI mice relative to WT CD4 cells (Fig. 4f; Supplementary Fig. 6). Other TCRβ rearrangements (for example, TRBV15) were not affected in any of the analysed GSK3β-KI mice (Fig. 4g; Supplementary Fig. 6). In addition, we performed quantitative analysis of a panel of Vβ with Jβ1 and Jβ2 rearrangements using genomic DNA from WT and GSK3β-KI DN thymocytes^30^. The frequency of TRBV19 with Jβ2 rearrangement was lower in GSK3β-KI DN thymocytes (Fig. 4h), but there was no statistically significant difference for the frequency of TRBV19 with Jβ1 rearrangement. The TRBV16-Jβ2 rearrangement frequency was also reduced in GSK3β-KI thymocytes, while the frequency of TRBV16-Jβ1 was not significantly different, although a trend was observed (Fig. 4h). The frequency of the other examined rearrangements of Vβ segment with Jβ1 or Jβ2 were not significantly affected (Supplementary Fig. 7). Thus, the inactivation of GSK3β by phosphorylation on Ser^389^ promotes the survival of DN3 thymocytes during V(D)J recombination of the preselection TCRβ locus. Failure to inactivate GSK3β through this pathway diminishes DN3 thymocyte fitness and success in recombining the TCRβ locus leading to an altered TCRβ repertoire.

### *In vivo* antibody response in GSK3β Ser^389^KI mice is impaired

**Figure 5:**
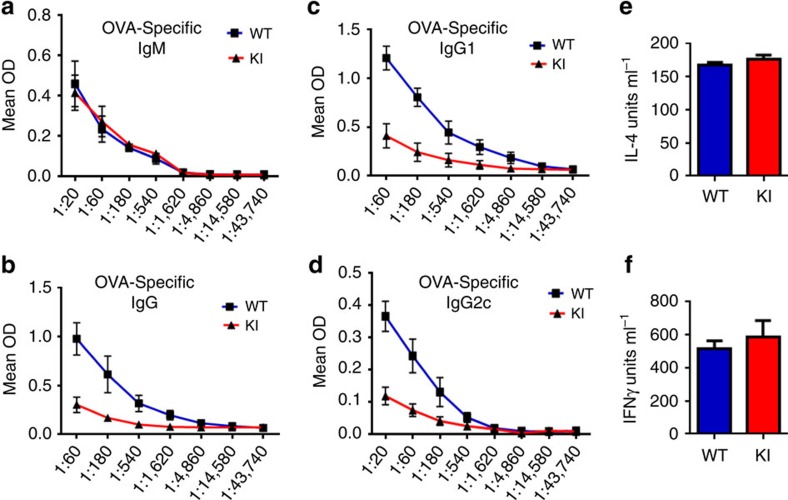
T-cell dependent antibody response *in vivo* requires phosphorylation of GSK3β at Ser^389^. WT and GSK3β-KI mice (*n*=4 mice per genotype) were immunized with OVA and CFA. After 14 days, mice were euthanized and serum was collected and OVA-specific IgM (**a**), IgG (**b**), IgG1(**c**) and IgG2c (**d**) were determined by ELISA. (**e**,**f**) Splenocytes were isolated from immunized WT and GSK3β-KI mice and restimulated *ex vivo* with OVA antigen. Production of IL-4 (**e**) and IFNγ (**f**) by were determined by ELISA. Data are shown as mean±s.e.m. and are representative of three independent experiments.

Since GSK3β phosphorylation on Ser^389^ was induced in B cells by CSR-generated DSBs, we investigated the effect of inactivation of GSK3β through this pathway on the *in vivo* humoral response. The percentage and the number of B cells in GSK3β-KI and WT mice was comparable (Supplementary Fig. 8a,b). No significant difference was detected in the baseline levels of total IgM or IgG1 (Supplementary Fig. 8c,d). To examine the effectiveness of CSR in an antigen-specific immune response, WT and GSK3β-KI mice were immunized with ovalbumin (OVA). After 14 days, immunized mice were euthanized and serum collected to assay antibody responses. The level of serum OVA-specific IgM was comparable in both WT and GSK3β-KI mice (Fig. 5a), as expected considering that production of IgM isotype-Abs does not require CSR. In contrast, the production of OVA-specific IgG, which requires CSR, was largely abrogated in GSK3β-KI mice (Fig. 5b). Moreover, IgG isotype analysis revealed reduced OVA-specific IgG1 (Fig. 5c) and OVA-specific IgG2c (Fig. 5d) in GSK3β-KI mice. Together, these results show for the first time that inactivation of GSK3β by phosphorylation of Ser^389^ is essential for efficient CSR and antibody production *in vivo*.

Since cytokines derived from CD4 cells play an important role in promoting isotype switching of B cells, the impairment in IgG production could be due to an impaired cytokine production rather than to an intrinsic defect in B cells. To address this possibility splenocytes from immunized WT and GSK3β-KI mice were restimulated *ex vivo* with OVA, and the levels in the supernatant of IL-4 and IFNγ, two major cytokines that promote IgG switching, were determined. The levels of both cytokines in WT and GSK3β-KI cells were comparable (Fig. 5e,f), indicating that T-cell helper responses are not impaired in GSK3β-KI mice. Thus, the failure to undergo *in vivo* class switching and to mount an antibody immune response in GSK3β-KI mice was most likely due to an intrinsic defect in B cells due to inability to inactivate nuclear GSK3β.

### Phospho-Ser^389^ GSK3β promotes fitness in response to CSR

**Figure 6:**
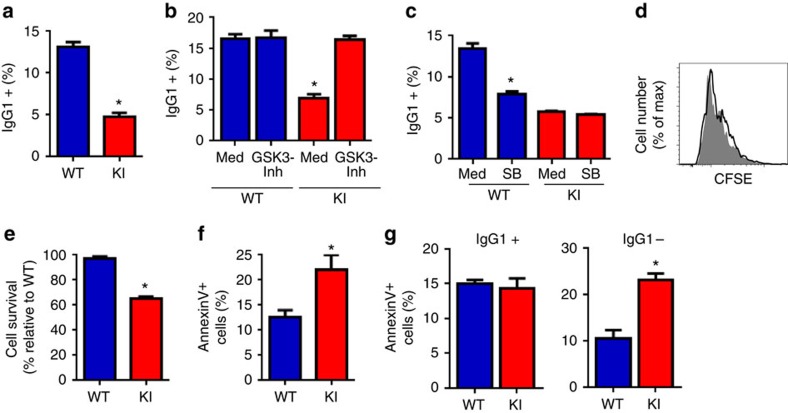
Inactivation of GSK3β by p38 MAPK-mediated phosphorylation on Ser^389^ is required for B cells to survive the DNA damage response triggered by CSR. (**a**) Purified B cells from WT and GSK3β-KI mice (*n*=3) were activated for four days with LPS and IL-4 and the surface level of IgG1 determined by flow cytometry. (**b**) B cells from WT and GSK3β-KI mice (*n*=3) were activated for 4 days either in the presence or absence of the GSK3 inhibitor X (GSK-Inh) and the percent of IgG1 positive B cells was determined as in **a**. (**c**) B cells from WT and GSK3β-KI mice (*n*=3) were activated as described above either in the presence or absence of the p38 MAPK inhibitor SB203580 (SB) and the percent of IgG1 positive B cells was determined as in **a**. (**d**) B cells from WT (solid black line) and GSK3β-KI (grey filled) mice (*n*=3) were loaded with CFSE and activated for four days and CFSE staining was examined flow cytometry analysis. (**e**) B cells from WT and GSK3β-KI mice (*n*=4) were activated for three days and viability determined by trypan blue staining. Percentage of cell survival relative to activated WT B cells is provided (% relative to WT cells). (**f**) B cells from WT and GSK3β-KI mice (*n*=4) were activated for three days and cell death was measured by AnnexinV staining and flow cytometry analysis. Percentage of AnnexinV positive cells for each cell type is shown. (**g**) B cells from WT and GSK3β-KI mice (*n*=3) were activated for three days and stained for AnnexinV, B220 and IgG1. The percentage of AnnexinV+ cells in the B220+IgG+ and B220+IgG- populations are shown. *P value<0.05 as determined by *t*- test. Data are shown as mean±s.e.m. and are representative of three or more independent experiments.

To demonstrate that the GSK3β Ser^389^Ala mutation in KI mice interferes with efficient CSR, we examined Ab production in isolated B cells activated with LPS in the presence of IL-4 *in vitro*. After 4 days, we examined the presence of IgG1 on the cell surface of B cells, as a parameter for CSR. The frequency of IgG1-expressing B cells from GSK3β KI mice was markedly reduced (Fig. 6a). Thus, phosphorylation of GSK3β on Ser^389^ is important for B cells undergoing CSR to successfully express cell surface IgG1. To demonstrate that the low frequency of IgG1-expressing B cells from GSK3β KI mice was due to the inability of these cells to inactivate GSK3β during CSR, B cells from WT and GSK3β-KI mice were activated in the presence or absence of the pharmacological inhibitor of GSK3 (GSK3 Inhibitor X). The GSK3 inhibitor had no effect on the generation of IgG1-expressing B cells from WT mice (Fig. 6b), supporting the existence of endogenous mechanisms that suppress GSK3β during CSR. Interestingly, the GSK3 inhibitor fully restored the frequency of IgG1-expressing B cells from GSK3β-KI mice (Fig. 6b). In addition, inhibition of p38 MAPK by SB203580 significantly reduced the frequency of IgG1-expressing WT B cells but it had no effect on the frequency of IgG1-expressing B cells from the GSK3β-KI mice (Fig. 6c) indicating that the effect of p38 MAPK on CSR in B cells is mediated through Ser^389^ phosphorylation. Thus, inactivation of GSK3β through the Ser^389^ phosphorylation by p38 MAPK is important for successful Ab isotype switching.

To determine whether the reduced frequency of IgG1-producing B cells was due to impaired expansion, we performed carboxyfluorescein succinimidyl ester (CFSE) labelling. No difference in proliferation was observed between WT and GSK3β-KI B cells (Fig. 6d). Similarly, there was no difference in cell cycle profiles between WT and GSK3β-KI B cells following activation (Supplementary Fig. 9). However, there was a significant reduction in survival of GSK3β-KI B cells relative to WT B cells based on cell recovery (Fig. 6e). Annexin V staining confirmed increased cell death in activated GSK3β-KI B cells (Fig. 6f; Supplementary Fig. 9b). Increased death in activated GSK3β-KI B cells was also supported by analysis of cell size (Supplementary Fig. 9c) and live/dead blue staining (Supplementary Fig. 9d,e). Together, these results show that GSK3β Ser^389^Ala mutation decreases the fitness of B cells while undergoing CSR, compromising their ability to produce antibodies.

### Mcl-1 degradation causes necroptosis in KI B cells

**Figure 7:**
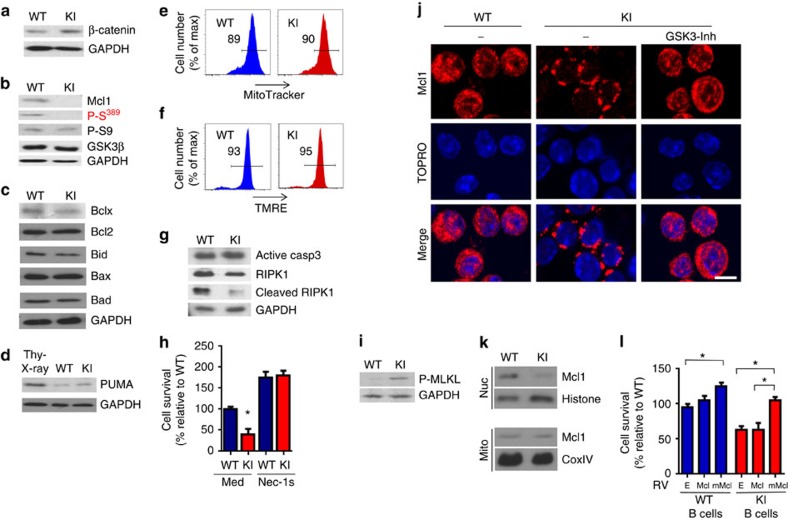
Inactivation of GSK3β by p38 MAPK promotes accumulation of the prosurvival factor Mcl-1. (**a**,**b**) WT and GSK3β-KI B cells were activated for two days and the expression of β-catenin (**a**), Mcl-1, P-S^389^ GSK3β and total GSK3β (**b**) were examined by western blotting. GAPDH is shown as a loading control. (**c**) WT and GSK3β-KI B cells were activated as in **a** and Bclx, Bcl2, Bid, Bax and Bad levels were examined by western blotting. (**d**) Western blot analysis for PUMA in irradiated WT thymocytes (5 Gy) and activated WT and GSK3β-KI B cells. (**e**,**f**) B cells activated as in **a** were stained with MitoTracker (**e**) or TMRE (**f**) and analysed by flow cytometry. The number represents the percentage of cells within the gate. (**g**) WT and GSK3β-KI B cells were examined by western blotting for the expression of cleaved caspase-3 (activated caspase-3), full length RIPK1 (RIPK1) and cleaved RIPK1 (cleaved RIPK1). (**h**) WT and GSK3β-KI B cells were activated, after 2 days Nectrostatin-1s (Nec-1s) was added and cell viability was determined by cell counting 24 h later (*n*=3). (**i**) WT and GSK3β-KI B cells were activated for 3 days and phospho-MLKL was examined by western blotting. (**j**) WT and GSK3β-KI B cells in the presence or absence of the GSK3 inhibitor (GSK3-Inh) were activated and examined by immunostaining and confocal microscopy for Mcl-1 (red) and TOPRO nuclear stain (blue). Scale bar, 3 μm. (**k**) Mcl-1 levels in nuclear (Nuc) and mitochondrial (Mito) extracts from activated WT and GSK3β-KI B cells were determined by western blot analysis. Histone and CoxIV (Complex IV) were used as loading controls. (**l**) WT and GSK3β-KI B cells were transduced with either an empty retrovirus (E), a retrovirus expressing wildtype Mcl-1 (Mcl) or a retrovirus expressing a Ser^140^Ala mutant of Mcl-1 (mMcl). Three days after activation cell viability was determined by cell counting. (*n*=3) and *P value<0.05 as determined by *t*- test (**h**) or one-way ANOVA (**l**). Data are representative of three or more independent experiments.

One of the mechanisms by which GSK3β can mediate cell death is by phosphorylation of β-catenin in the cytosol, leading to the rapid degradation of β-catenin by APC complex^31^,^32^. However, β-catenin levels were comparable in activated GSK3β-KI B cells and WT B cells (Fig. 7a). GSK3β also promotes the degradation of Mcl-1 through phosphorylation on Ser^159^ (mouse Ser^140^)^33^. In response to DSBs, Mcl-1 accumulates in the nucleus, where it associates with γH2AX and DSB^34^,^35^, and it is required for DSB response through different mechanisms^35^. Mcl-1 deficiency results in increased genomic instability and it is essential for B-cell survival following activation^34^,^35^,^36^. Interestingly, western blot analysis showed that the levels of Mcl-1 were almost undetectable in activated GSK3β-KI B cells (Fig. 7b). To rule out that the reduced levels of Mcl-1 were not the result of increased mitochondrial dysfunction in GSK3β-KI B cells we examined the levels of other Bcl2 family members. The levels of Bcl2, BclX (anti-apoptotic) as well as Bid, Bax and Bad (pro-apoptotic) were not different between WT and GSK3β-KI B cells (Fig. 7c). Mcl-1 has been shown to regulate PUMA^37^ that also mediates apoptosis in response to DNA damage^38^. PUMA was highly expressed in thymocytes exposed to irradiation (Fig. 7d), but only marginal levels could be detected in either WT or GSK3β-KI B cells (Fig. 7d). We also examined mitochondria integrity by staining with Mitotracker (Fig. 7e), as well as mitochondrial membrane potential by staining with TMRE (Fig. 7f), but no differences between WT and GSK3β-KI B cells were detected. The reduction of Mcl-1 in GSK3β-KI B cells therefore was not associated with dysfunctional mitochondria.

These results also suggested that increased death found in GSK3β-KI B cells might not be through apoptosis mediated by mitochondria and caspase-3. Analysis of caspase-3 activation showed comparable levels of active caspase-3 (cleaved form) in activated WT and GSK3β-KI B cells (Fig. 7g). In addition, most of the Annexin V-positive cells were propidium iodide positive (Supplementary Fig. 10), suggesting the presence of other death pathways. DNA damage has also been shown to trigger necroptosis, a mitochondria-independent mechanism of death^39^,^40^. RIPK1, the most upstream component of the ripoptosome pathway, is normally cleaved to prevent the formation of the necrosome and death by necroptosis^41^. Cleaved RIPK1 was present in activated WT B cells, but only low levels could be detected in GSK3β-KI B cells (Fig. 7g). *In vitro*, blocking of caspase-8 promotes death by necroptosis^42^. Negligible levels of cleaved caspase-8 were detected in both activated WT and GSK3β-KI B cells (Supplementary Fig. 11a), suggesting that there is minimal activity of caspase-8 in activated B cells overall. As a positive control for cleaved caspase-8 we used activated B cells treated with Fas ligand known to trigger caspase-8 cleavage (Supplementary Fig. 11a). Thus, the increased death found in activated B cells that fail to inhibit GSK3β could be mediated by necroptosis. We therefore examined the effect of necrostatin-1s, a selective inhibitor of necroptosis through RIPK1 (ref. 43). WT and GSK3β-KI B cells were activated. Necrostatin-1s was added after 2 days of activation, when DSBs and phospho-Ser^389^ GSK3β were detected (Fig. 3) and before differences in survival were observed (Supplementary Fig. 11b). Cell survival was determined 24 h after treatment with necrostatin-1s (day 3 of activation). Interestingly, treatment with necrostatin-1 restored survival of activated GSK3β-KI B cells (Fig. 1h) Similar results were obtained adding necrostatin-1s at 24 and 36 h following activation (Supplementary Fig. 11c). During necroptosis active RIPK1 activates the downstream kinase RIPK3, and RIPK3 then mediates the phosphorylation of MLKL, one of the mediators of necroptosis that translocates to the membrane, causes membrane rupture and death^40^,^44^,^45^. Increased levels in phospho-MLKL were detected in GSK3β-KI B cells (Fig. 7i). Thus, inactivation of nuclear GSK3β by phosphorylation on Ser^389^ is a mechanism to minimize necroptosis-mediated cell death of B cells during CSR.

Since the subcellular localization of Mcl-1 in activated B cells has not been addressed, we performed immunostaining for Mcl-1 and confocal microscopy analysis. Abundant levels of Mcl-1 were present both in the cytosol and the nucleus of WT B cells (Fig. 7j). In contrast, the remaining Mcl-1 levels in GSK3β-KI B cells were almost exclusively outside the nucleus (Fig. 7j). Treatment of GSK3β-KI B cells with the GSK3 inhibitor restored Mcl-1 levels in the nucleus to those in WT B cells (Fig. 7j). In addition, western blot analysis for Mcl-1 showed comparable levels in the mitochondrial fraction, however, levels of Mcl-1 in the nuclear fraction were drastically decreased in GSK3β-KI B cells (Fig. 7k). To address whether the decreased nuclear levels of Mcl-1 contribute to the impaired survival of GSK3β-KI B cells we ectopically expressed WT Mcl-1, or a previously described mutant form of Mcl-1 (mMcl-1) where Ser^140^ is replaced by Ala (Ser^140^Ala)^46^ and therefore it cannot be phosphorylated by GSK3β and degraded^33^,^46^. Thus, WT Mcl-1 will still be degraded by GSK3β Ser^389^Ala in KI B cells, but the mMcl-1 mutant should be protected from degradation. Accordingly, WT Mcl-1 expression did not increase survival of GSK3β-KI B cells relative to empty vector (Fig. 7l). In contrast, expression of Ser^140^Ala mMcl-1 restored survival of GSK3β-KI B cells (Fig. 7l). Efficiency of transduction with both retroviruses in GSK3β-KI B cells was comparable (Supplementary Fig. 12a), and the total levels of Mcl-1 expression as determined by qRT–PCR (quantitative PCR with reverse transcription) was also comparable (Supplementary Fig. 12b). Therefore, nuclear Mcl-1 present in activated B cells is selectively targeted by GSK3β for degradation, and inactivation of GSK3β through phosphorylation on Ser^389^ is necessary to sustain Mcl-1 levels and fitness of B cells undergoing CSR.

## Discussion

DSBs are dangerous types of DNA damage because they create genomic instability and impair cell survival. However, V(D)J and CSR-mediated DSBs are essential for generating the diversity of our adaptive immune system. Here we identify phosphorylation of GSK3β at Ser^389^/Thr^390^ as a survival pathway that is triggered specifically by DSBs, in contrast to Ser^9^ phosphorylation that is used as a general mechanism to restrict GSK3β activity. In addition, we show that Ser^389^/Thr^390^ phosphorylation regulates GSK3β in the nucleus where GSK3β associates with γH2AX in response to DSBs. The selective induction of Ser^389^ phosphorylation by DSBs could explain the selective tissue distribution of this phosphorylation, mainly in the thymus and spleen. Interestingly, phospho-Ser^389^ is also abundant in brain^18^ where recent studies have reported the presence of DSBs caused by normal brain activity and stress^47^. We show here that the inability of cells to inactivate GSK3β through Ser^389^ phosphorylation (GSK3β Ser^389^Ala mutant cells) causes a decline in the fitness of cells undergoing DSB induced by V(D)J recombination or CSR. We have also shown that failure to inactivate GSK3β through Ser^389^ phosphorylation in CD4 cells from GSK3β KI mice makes these cells more sensitive to death caused by low dose of irradiation (Supplementary Fig. 13a) or the chemotherapeutic drug doxorubicin (Supplementary Fig. 13b,c). Thus, phosphorylation of GSK3β on Ser^389^ in response to chemotherapy or radiotherapy may have implications in the treatment of cancer.

This study reveals a role of GSK3β in B cells. We demonstrate that inactivation of GSK3β by Ser^389^ phosphorylation upon activation of B cells is required optimum survival during CSR and for *in vivo* antigen-specific IgG antibody production. Suppression of p53 by BCL6 has been reported to be a prosurvival signal in germinal centre (GC) B cells^48^,^49^. In addition, both Mcl-1 (ref. 36) and c-Myc^50^ are also essential for GC B-cell survival although it is unclear how they are regulated. Our studies show that inactivation of nuclear GSK3β by phosphorylation at Ser^389^ is essential for the stabilization of the nuclear pool of Mcl-1. While Mcl-1 localizes predominantly in the mitochondria it is also found in the nucleus specifically in response to DSBs^34^,^35^. GSK3β has been shown to increase Mcl-1 instability through phosphorylation on Ser^140^ (ref. 46). Here we show that only Ser^140^Ala mutant Mcl-1, but not WT Mcl-1, restores viability of activated GSK3β-KI B cells. Thus, preventing phosphorylation of Mcl-1 on Ser^140^ by nuclear GSK3β could be a key mechanism to prolong survival during the repair of DSBs. Interestingly, our studies also reveal necroptosis as an important mechanism of death in B cells late during activation. DSBs are one of the reported stimuli that can promote necroptosis^51^,^52^. Inactivation of GSK3β through phosphorylation of Ser^389^ restrains necroptosis, promoting successful CSR and antibody production. Future studies will be needed to address if the crosstalk between GSK3β, Mcl-1 and necroptosis is also observed in other DSB responses.

Our studies show that inactivation of GSK3β by Ser^389^ phosphorylation contributes to the survival of DN3 thymocytes undergoing TCRβ rearrangement, leading to a reduction in the number of DN4 thymocytes. Similar phenotypes have been also described for mice deficient in ATM, Nbs1 γH2AX, that have impaired DSB repair responses^53^,^54^,^55^. A number of parameters such as chromatin environment influence the frequency of the TCRβ V-DJ rearrangements before TCRβ selection^30^. Impaired survival of thymocytes undergoing specific TCRβ rearrangements could also affect the TCRβ repertoire. Here we show decreased frequencies of some TCRβ rearrangements in GSK3β-KI mice. TCRβ repertoire is also impaired in the absence of ATM through an antigen selection-independent mechanism^54^. Our data suggest that inactivation of GSK3β by Ser^389^ is not essential for survival of DP thymocytes during TCRα recombination. Sequential recombination of D to J and DJ to V of the TCRβ locus may need additional survival signals. Likewise, the secondary recombination event in the TCRβ locus between V to DJ2, after an out of frame V to DJ1 rearrangement, may be more dependent on survival signals, as suggested by our results.

**Figure 8:**
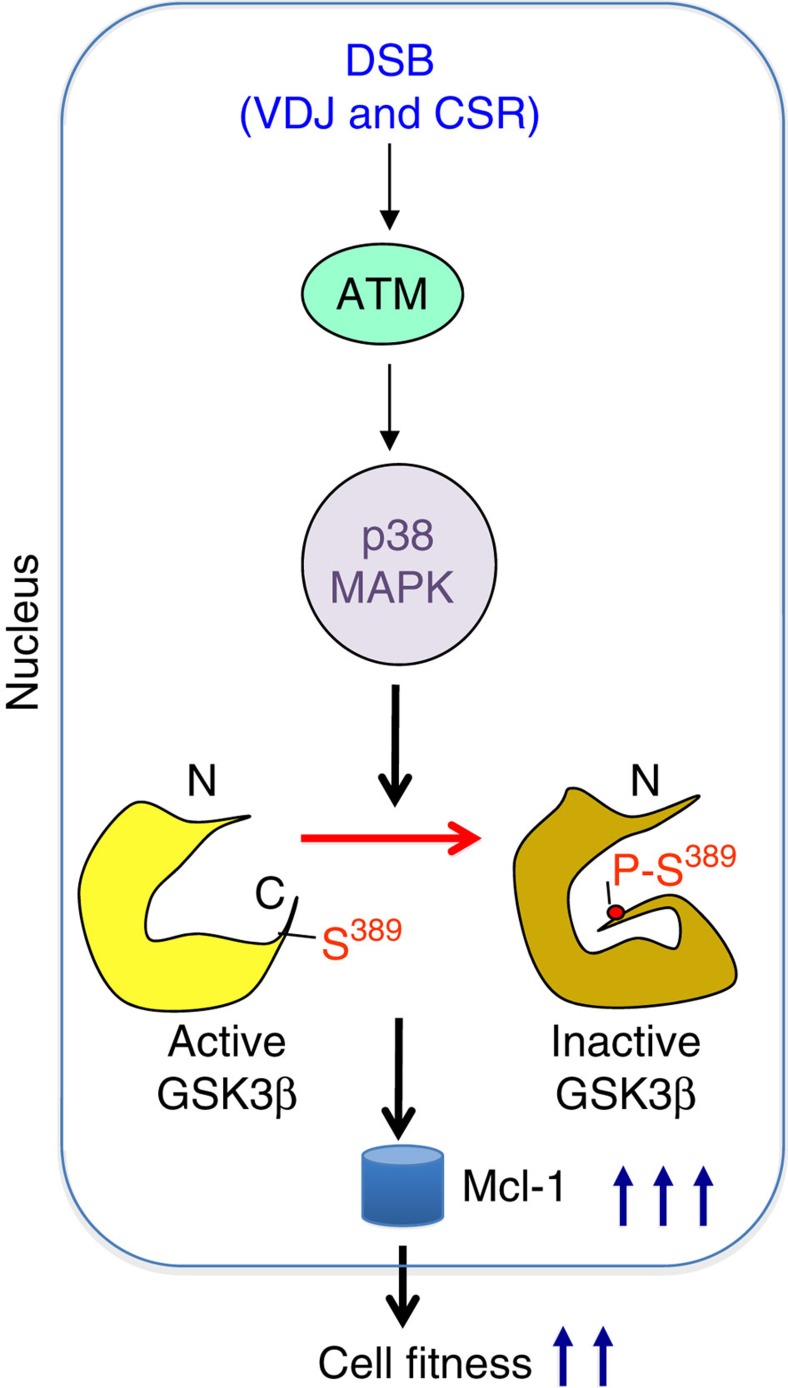
DSBs trigger the inactivation of GSK3β by p38 MAPK to promote the accumulation of the prosurvival factor Mcl-1 leading to enhanced cell fitness. Schematic model for the pathway.

In summary, our study reveals for the first time a specific pathway for regulation of nuclear GSK3β in response specifically to DSBs, including V(D)J recombination and CSR (Fig. 8). We also demonstrate that this inactivation pathway of GSK3β is essential for fitness of cells undergoing DSBs and therefore has an important role in the adaptive immune response.

## Methods

### Mice

For the generation of GSK3β KI mice, a targeting vector constructed (InGenious Targeting Laboratory, Inc.) from a 9.27 kb region subcloned from the C57BL/B6 BAC clone (RPCI-23: 454D5) into the vector backbone (2.4 kb pSP7 (Promega)). The long homology arm extended 6.36 kb 5′ to the T-G point mutation in exon 11 and the LoxP/FRT flanked Neo cassette was inserted 511 bp 3′ to the T-G point mutation. The short homology arm extended 2.40 kb 3′ to the loxP flanked Neo cassette. The targeting vector was constructed using Red/ET recombineering technology and the T-G point mutation was created by overlap PCR. The linearized targeting vector electroporated into BA1(C57BL/6 × 129/SvEv) hybrid embryonic stem cells. Following G418 selection, surviving clones were screened by PCR to identify homologous recombinant clones for injection into blastocysts. The neomycin cassette was excised by breeding heterozygous GSK3β KI mice with EIIa-cre mice^56^ (Jackson Laboratories). GSK3β KI mice were backcrossed to C57BL/6 at least seven generations. Wild-type C57BL/B6, 129S6/SvEvTac ATM KO (ref. 57), and B6.129S7 Rag1 KO (ref. 25) mice were purchased from Jackson Laboratories. C57BL/B6 p38α conditional knockout (p38 cKO) mice were generated by crossing the homozygous floxed p38α mice^58^ (Riken) with C57BL/B6 T-cell-specific Lck-Cre transgenic mice^59^ (Taconic). Balb/c AID KO mice were derived from AID KO mice^60^. For studies with DN3 thymocytes, 3–4-week old male and female mice were used. Male and female mice 8–16 weeks of age were used in all other experiments. Mice in each experiment were age and sex matched. All procedures that involved mice were approved by the University of Vermont's institutional guidelines for animal care.

### Human samples

Healthy volunteers and patients who agreed to donate peripheral blood signed a consent form that was approved by the Institutional Review Board of The University of Vermont. Breast cancer patients who were undergoing local radiation therapy as a first regimen of therapy and had not received any chemotherapy or hormonal based-therapy were enroled in the study. Blood was drawn before beginning radiotherapy treatment (baseline), and after the last of the four consecutive doses of the first cycle with radiotherapy (X-rad). CD4 T cells were purified from peripheral blood mononuclear cells by positive selection using the CD4 MACS kit (Miltenyi Biotec) as recommended by the manufacturer.

### X-ray exposure

For *in vivo* X-radiation (RadSource 2000), mice were exposed to the indicated dose and sacrificed following a 1.5 h recovery period. For X-irradiation of cells, cells were exposed to the indicated dose and harvested 18 h post exposure. Freshly isolated thymocytes were exposed to 5 Gy and allowed to recover for 1 h before harvest.

### Immunizations

Mice were immunized subcutaneously with OVA (Sigma) in CFA (Sigma) and euthanized after 14 days. Blood was collected and allowed to coagulate in the refrigerator in Microtainer Serum Separator tube (BD). Serum was isolated by centrifugation. For ELISA to detect OVA-specific Ig, plates were coated with 2 mg ml^−1^ OVA. Antibody levels in serially diluted serum samples were analysed with HRP-conjugated antibodies (1:4,000) specific for mouse IgM (1021-05), IgG (1030-05), IgG1 (1070-05), and IgG2c (1079-05) (SouthernBiotech) following manufacture's recommended protocols. For *ex vivo* cytokine production, splenocytes were isolated 14 days following OVA-CFA immunization and cultured in the presence of OVA (100 μg ml^−1^). ELISAs were performed with purified anti-IFNγ (Biolegend 505707) and anti-IL-4 mAb (Biolegend 504102) as capture antibodies, the corresponding biotinylated anti-IFNγ (Biolegend 505804, 1 μg ml^−1^)and anti-IL-4 mAb (BD Biosciences 554390 1 μg ml^−1^), horseradish peroxidase-conjugated streptavidin (Sigma), and the TMB microwell peroxidase substrate and stop solution (Kirkegaard & Perry Labs, Inc.) according to the recommended protocol (Biolegend). Recombinant mouse IFNγ and IL-4 (R&D Systems) was used as standards.

### Western blot analysis

Whole-cell extracts were prepared in Triton lysis buffer (20 mM Tris (pH 7.4), 137 mM NaCl, 2 mM EDTA, 1% Triton X-100, 10% glycerol, 25 mM β-glycerophosphate and 1 mM sodium vanadate) supplemented with mammalian protease inhibitor cocktail (Sigma). Nuclear and cytosolic extracts were prepared as follows: fresh cell pellet was resuspended in 400 μl cold buffer A (10 mM HEPES pH 7.9, 10 mM KCI, 0.1 mM, EDTA, 0.1 mM EGTA, 1 mM DTT and 0.5 mM PMSF) supplemented with mammalian protease inhibitor cocktail (Sigma). After 15 min on ice, 25 μl of a 10% solution of IGEPAL (Sigma) was added while vigorously vortexing for 10 s. The homogenate was centrifuged for 30 s at 13,000 r.p.m. The supernatant containing cytoplasm was transferred to a fresh tube. The nuclear pellet was resuspended in 50 μl ice-cold nuclear lysis buffer (20 mM HEPES pH 7.9, 0.4 M NaCl, 1 mM EDTA, 1 mM EGTA,1 mM DTT and 1 mM PMSF) supplemented with mammalian protease inhibitor cocktail (Sigma) and the tube is vortexed at 4 °C for 15 min. The nuclear extract was centrifuged for 5 min at 13,000 r.p.m. Mitochondrial extracts were prepared using the Mitochondrial Fractionation Kit (Active Motif).

Protein lysates were resolved by SDS–polyacrylamide gel electrophoresis and transferred to PVDF membrane (Millipore). All primary antibodies were used at 1:1,000. Anti-actin (SC-1616), anti-p38 MAPK (SC-7972), anti-GAPDH (SC-25778, anti-histone H1 (SC-8030), anti-PUMA (SC-377015) and anti-GSK3β (SC-9166) were purchased from Santa Cruz Biotechnology. Anti-phospho-p38 MAPK (#9215), anti-γH2AX(#2577), anti-CoxIV (#4844), anti-Caspase-3 (#9662), and anti-phospho-Ser^9^ GSK3β (#9323) were purchased from Cell Signaling. Anti-Bcl2 (B46620), anti-Bclx (B61220), anti-Bid (B21320), anti-Bax (B73520), anti-Bad (B36420) and anti-RIPK1 (610459) were purchased from BD Biosciences. Anti-Mcl (600-401-394) was purchased from Rockland. Anti-phospho-MLKL (196436) was purchased from Abcam. Anti-Caspase-8 (ALX-804-488-C100) was purchased from Enzo. Anti-phospho-Ser^389^ was previously described^18^. Anti-phospho-Thr^390^ GSK3β rabbit polyclonal antibody was generated (Proteintech, Inc.) using N-C-ARIQAAASphospho-TPTNATA for immunization. Anti-rabbit HRP (Jackson ImmunoResearch Laboratories 111-035-144, 1:10,000), anti-mouse HRP (Jackson ImmunoResearch Laboratories 115-035-166, 1:10,000), anti-rat HRP (Dako P0450, 1:1,000) and anti-goat HRP (Santa Cruz Biotechnology SC-2056, 1:10,000) were used as secondary antibodies. For quantitative western blot analysis of human samples, secondary IRD-tagged antibodies (800 IRDye and 680 IRDye; Li-COR) were used and western blot signals were analysed with an Odyssey Li-COR laser scanning and imaging system (Li-COR, v3.0). Images have been cropped for presentation. The uncropped images are shown in the Supplementary Figs 15–18.

### GSK3β kinase activity assays

GSK3β was immunoprecipitated from the indicated protein lysates with anti-GSK3β (Cell Signaling #9315) and protein A/G PLUS agarose beads (Santa Cruz SC2003). For kinase assays, reactions were incubated at 30 °C in kinase buffer (Cell Signaling) supplemented with 1 mM ATP containing [γ-32P] ATP and GSM substrate peptide (62.5 μM) (Upstate). Reactions were terminated by spotting onto P81 filters. Filters were washed extensively and counted in a scintillation counter.

### Cell culture

MCF-7 cells were maintained in RPMI (Lonza) supplemented with 5% FBS, L-glutamine, penicillin and streptomycin (Invitrogen). Cells were stimulated with doxorubicin (0.1 μg ml^−1^) (Sigma) for 18 h. For all inhibitor treatments, SB203580 (5 μM), ATM inhibitor (5 μM) and GSK3 inhibitor X (2.5 μM; Calbiochem) pretreatment was performed at 37 °C for 30 min, before cell stimulation. CD4 T cells were isolated from spleens by positive selection using anti-CD4 magnetic beads (Miltenyi). The purity obtained was about 90–95% (Supplementary Fig. 14A). CD4 cells were treated with doxorubicin (0.1 μg ml^−1^) or stimulated with plate bound anti-CD3 (5 μg ml^−1^) and soluble anti-CD28 (1 μg ml^−1^) mAbs (BD Biosciences) for 18 h before analysis. B cells were isolated from spleens by positive selection with anti-CD19 magnetic beads (Miltenyi). Purity for B cells obtained using this approach was 95% (Supplementary Fig. 14B). B cells were stimulated with LPS (25 μg ml^−1^; Sigma) and recombinant mouse Il-4 (10 ng ml^−1^; PeproTech) for the indicated period of time. Necrostatin-1s (10 μM; BioVision) was added at the indicated time following activation. After 2 days of activation, B cells were treated for 3 h with human recombinant flag-tagged Fas Ligand (200 ng ml^−1^) and anti-Flag antibody (2 μg ml^−1^; Sigma) for caspase-8 activation positive control. Recombinant retroviruses were prepared by transient transfection of plasmids in 293T cells. B cells activated for 24 h were transduced with retrovirus produced using either pBABE-IRES-Puro empty vector, wt Mcl-1 or a mMcl-1 Ser140Ala expression vector. Retroviral transduction efficiency was examined by real-time qPCR using equal amount of DNA isolated from transfected cells and measuring retroviral vector Mcl-1 DNA. Total mRNA Mcl-1 levels were determined by real-time qRT–PCR using total RNA from transduced cells and 18S expression as housekeeping gene. Taqman Mcl-1 AOD (assay on demand) primers/probe set was used for both qPCR and qRT–PCR (Applied Biosystems).

### Flow cytometry

Thymocyte and T-cell subpopulations were examined after cell surface staining with anti-CD4 (Biolegend 100530, 1:200), CD8 (Invitrogen MCDO817, 1:200), CD25 (Biolegend 102006, 1:200) and anti-CD44 antibodies (BD Biosciences 559250, 1:200) by flow cytometry (LSR II, BD Biosciences). DN3 and DP cells were sorted after four colour staining (FacsAria, BD Biosciences). DN cells were obtained by depletion of CD4- and CD8-positive cells using magnetic beads (Qiagen). Thymocytes were stained for surface antigens and then permeabilized with 0.3% saponin to allow detection of intracellular TCRβ (Biolegend 109222, 1:200). Cells from spleen, lymph nodes and purified B cells were treated with FC Block (BD Biosciences 553141, 1:100) before staining surface antigens. Cell surface IgG1 was analysed by flow cytometry after surface staining with anti-B220 (553088, 1:200) and anti-IgG1 (553441, 1:200; BD Biosciences). Annexin V (Biolegend) staining with and without propidium iodine (Sigma) was performed as recommended by the manufacturer. CFSE (Molecular Probes) labelling was performed according to the manufacturer's instructions. Cells (1 × 10^7^ cells per ml) were incubated with 2.5 μM CFSE in 0.05% BSA in PBS at 37 °C for 10 min and activated to stimulate proliferation. For cell cycle analysis, cells (10^6^ cells) were resuspended in low salt staining solution (3% (w/v) polyethylene glycol PEG 8000, 50 μg ml^−1^ propidium iodide, 180 U ml^−1^ RNase A, 0.1% Triton X-100, 4 mM sodium citrate) and incubated on ice for 30 min. An equal volume of high salt solution (3% (w/v) polyethylene glycol PEG 8000, 50 μg ml^−1^ propidium iodide, 180 U ml^−1^ RNase A, 0.1% Triton X-100 and 400 mM sodium chloride) was added just before analysis by flow cytometry. Mitochondrial functional analysis was performed by staining with MitoTracker (Life Technologies) or TMRE (Molecular Probes) for 20 min at 37 °C as recommended by the manufacturer.

### Confocal microscopy

For staining of MCF-7 cells and lymphocytes, cells were fixed, permeabilized and stained with primary antibody followed by fluorescently labelled secondary antibody. The primary antibodies used included the rabbit anti-phospho-Ser^389^ (1:500) or anti-phospho-Thr^390^ (1:250) GSK3β and anti-Mcl-1 (1:200) polyclonal antibodies described above. Mouse monoclonal anti-γH2AX (Millipore 05–636, 1:300) was used for co-staining with phospho-S^389^ GSK3β. Secondary antibodies used were Alexa Fluor 568 anti-rabbit (A11036, 1:500) and Alexa Fluor 488 anti-mouse (A11029, 1:500) highly adsorbed antibodies (Invitrogen). Topro-3 (Invitrogen) was used as a nuclear stain. Images of fixed cells were acquired on a Zeiss LSM-510.

### *Tcrβ* sprectrotyping

CD4 T cells were isolated from spleens by positive selection using anti-CD4 magnetic beads (Miltenyi). Spectrotyping was carried out using the *SuperTCRExpress* CDR3 Size Diversity Determination Kit as per manufactures' instructions (BioMed Immunotech).

### *Tcrα* rearrangement

*Tcr*α rearrangement was analysed by real-time PCR using a QuantiFast SYBR Green PCR kit (Qiagen) and a Roche LightCycler 480. Primers are listed in (Supplementary Table 1). PCR reactions for individual experiments were run in duplicate using the following amplification program: 95 °C for 5 min, followed by 45 cycles of 95 °C for 10 s and 62 °C for 30 s. Results were normalized to signals for the *Tcr*α enhancer (E_α_).

### *Tcrβ* rearrangement

*Tcr*β rearrangement was analysed by real-time PCR using PerfeCta SYBRGreen SuperMix (Quanta Biosciences) and AB 7500 Fast Real-time PCR System (Applied Biosystems). Primers are listed in (Supplementary Table 2). PCR reactions for individual experiments were run in duplicate using the following amplification program: 95 °C for 2 min, followed by 40 cycles of 95 °C for 15 s, 60 °C for 1 min and 72 °C for 1 min. Results were normalized to signals for the *MAPK14* gene.

### Statistical analysis

Statistical significance between two groups was determined using Prism (Graphpad), by standard Student *t*-test. Statistical significance among more than two groups was determined by one-way ANOVA. For all analyses, *P*≤0.05 was considered significant.

## Additional information

**How to cite this article:** Thornton, T. M. *et al*. Inactivation of nuclear GSK3β by Ser^389^ phosphorylation promotes lymphocyte fitness during DNA double-strand break response. *Nat. Commun.* 7:10553 doi: 10.1038/ncomms10553 (2016).

## Supplementary Material

Supplementary Information Supplementary Figures 1-18 and Supplementary Table 1 and 2
