# Supplementary material for: Inactivation of nuclear GSK3β by Ser389 phosphorylation promotes lymphocyte fitness during DNA double-strand break response
Source: Nat Commun. 2016 Jan 29;7:10553. doi: 10.1038/ncomms10553 (PMC4740185; doi:10.1038/ncomms10553)
Supplement: Supplementary Information — Supplementary Figures 1-18 and Supplementary Table 1 and 2 [file ncomms10553-s1.pdf]

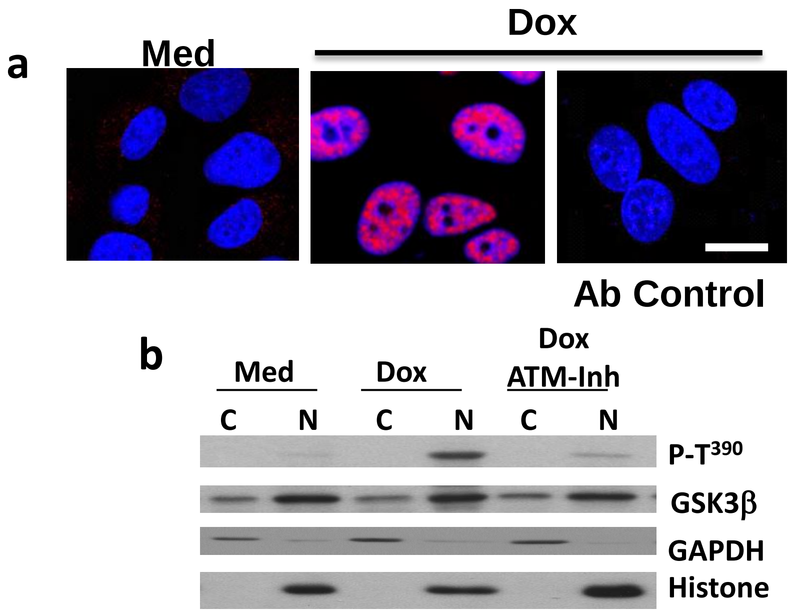

Supplementary Figure 1. (**a**) The presence of P-T^{390} GSK3β (red) and TOPRO nuclear staining (blue) in MCF7 cells treated with media (Med) or doxorubicin (Dox) were examined by immunostaining and confocal microscopy. Dox treated cells not stained for P-T^{390} GSK3β primary Ab are shown as a control for background staining. White scale bar=20μm (**b**) Western blot analysis for P-T^{390} GSK3β and total GSK3β using nuclear (N) and cytosolic (C) extracts from MCF7 cells treated with media (Med) or doxorubicin (Dox) in the presence or absence of the ATM inhibitor (ATM-Inh). GAPDH and histone are shown as controls.

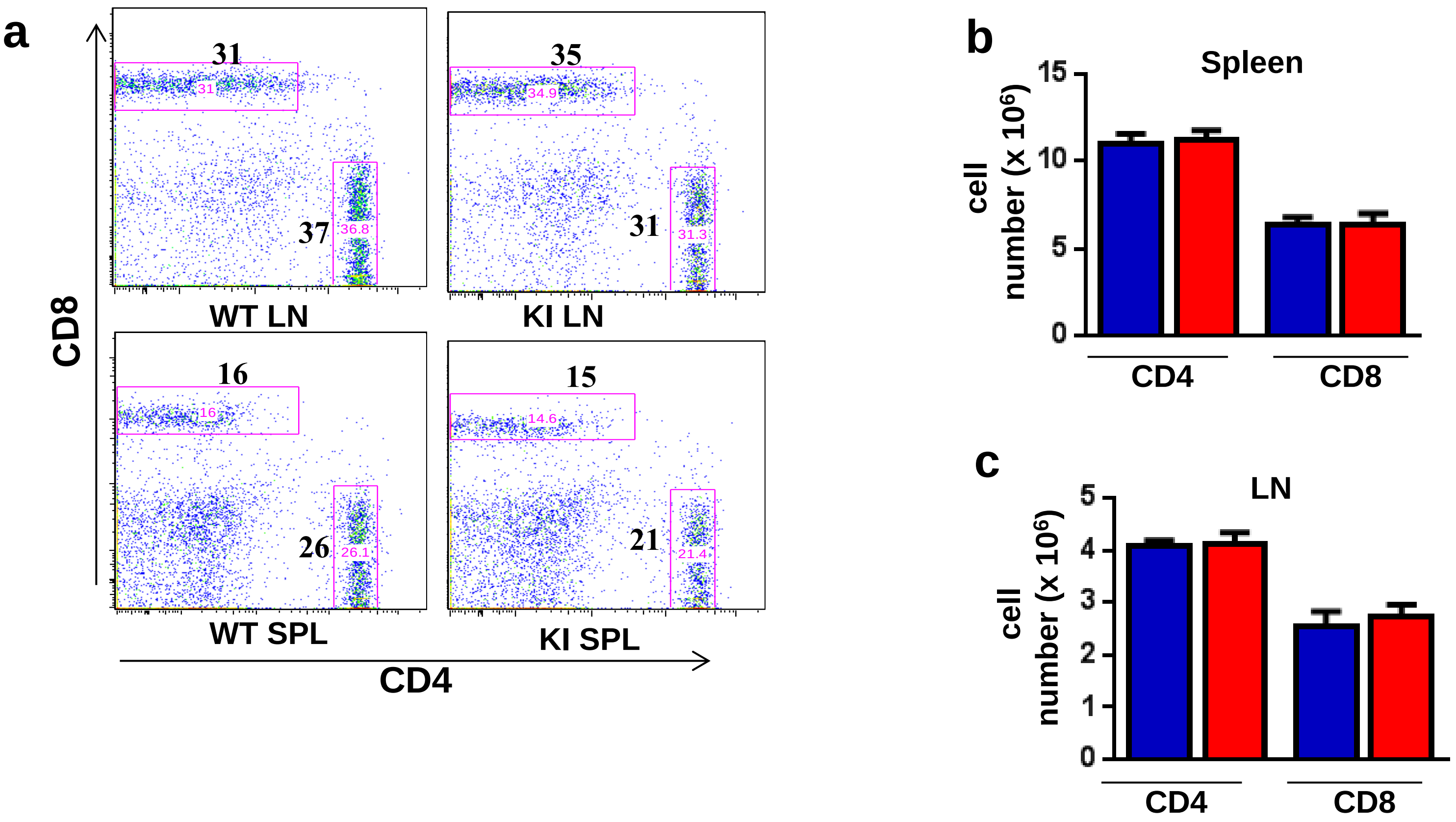

Supplementary Figure 2. (**a**) Representative FACS plots showing the percentages of CD4 and CD8 cells in the lymph nodes and spleens from WT and GSK3β KI mice. (**b** and **c**) The number of CD4 cells and CD8 cells in spleen (**b**) and lymph nodes (**c**)from WT and GSK3β KI mice (n=3).

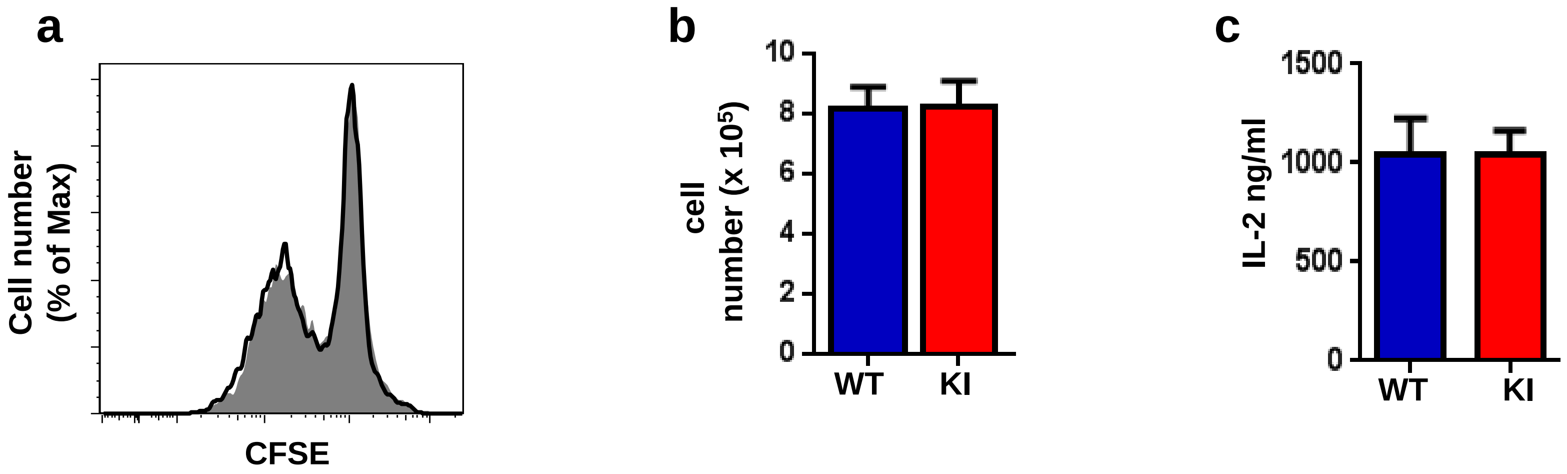

Supplementary Figure 3. (**a**) CD4 cells were isolated from WT (solid black line) and GSK3β KI (Gray filled) mice, labeled with CFSE and after three days of stimulation with anti-CD3 and anti-CD28, CFSE dilution was measured by flow cytometry. (**b**) Number of viable CD4 cells recovered after 3 days of activation as in (**A**) was determined by trypan blue staining and counting cells. (**c**) IL-2 production by WT and GSK3β KI CD4 cells following three days of activation.

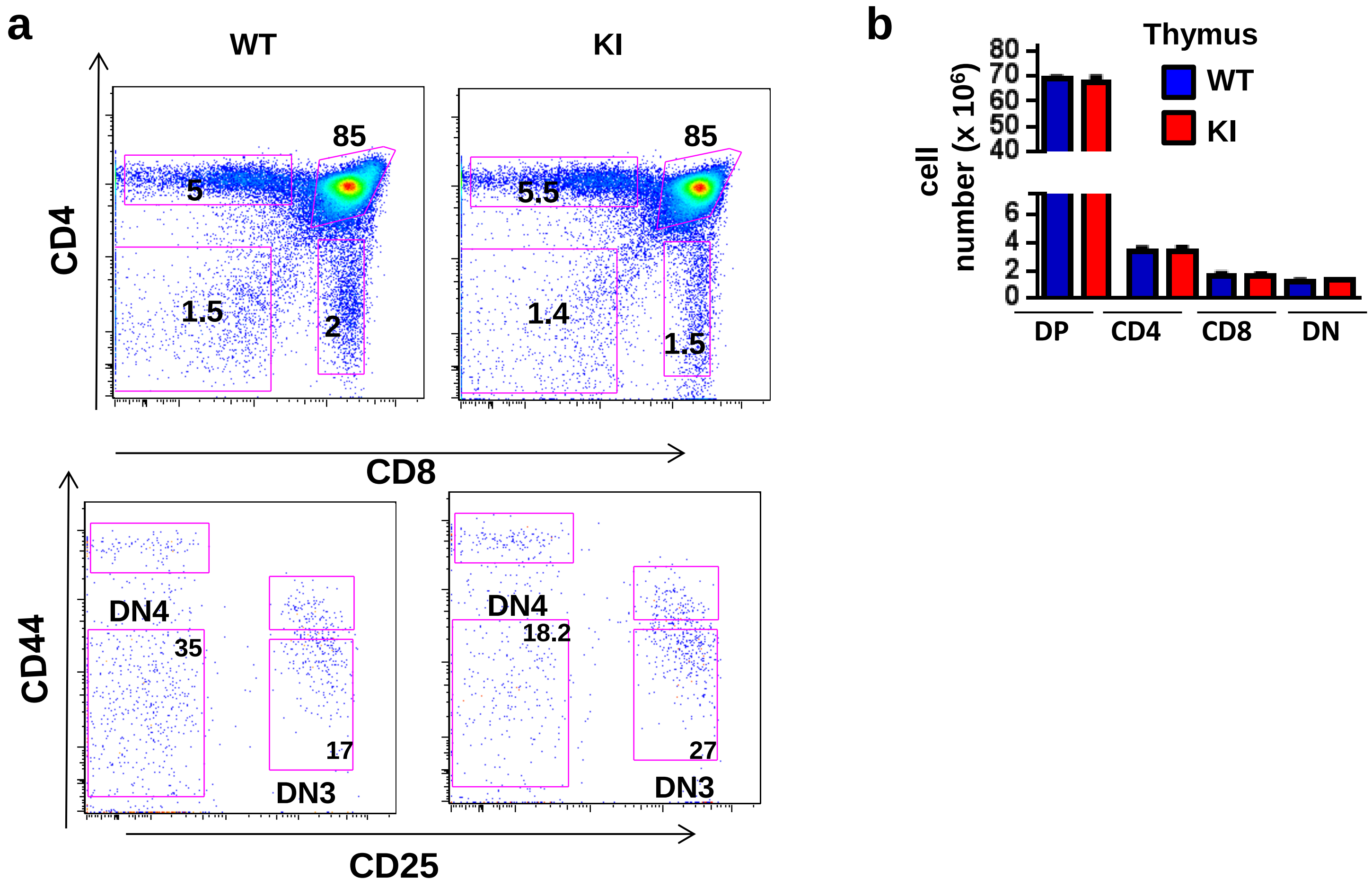

Supplementary Figure 4. (**a**) Representative FACS plots to show T cell development in the thymus from WT and GSK3β-KI mice. Double negative thymocytes were selected and CD44 and CD25 expression examined to determine DN3 and DN4 populations. The numbers represent the percentage of the cell population indicated. (**b**) Number of DP, CD4, CD8 and DN sub-populations of thymocytes from WT and GSK3β KI mice (n=8).

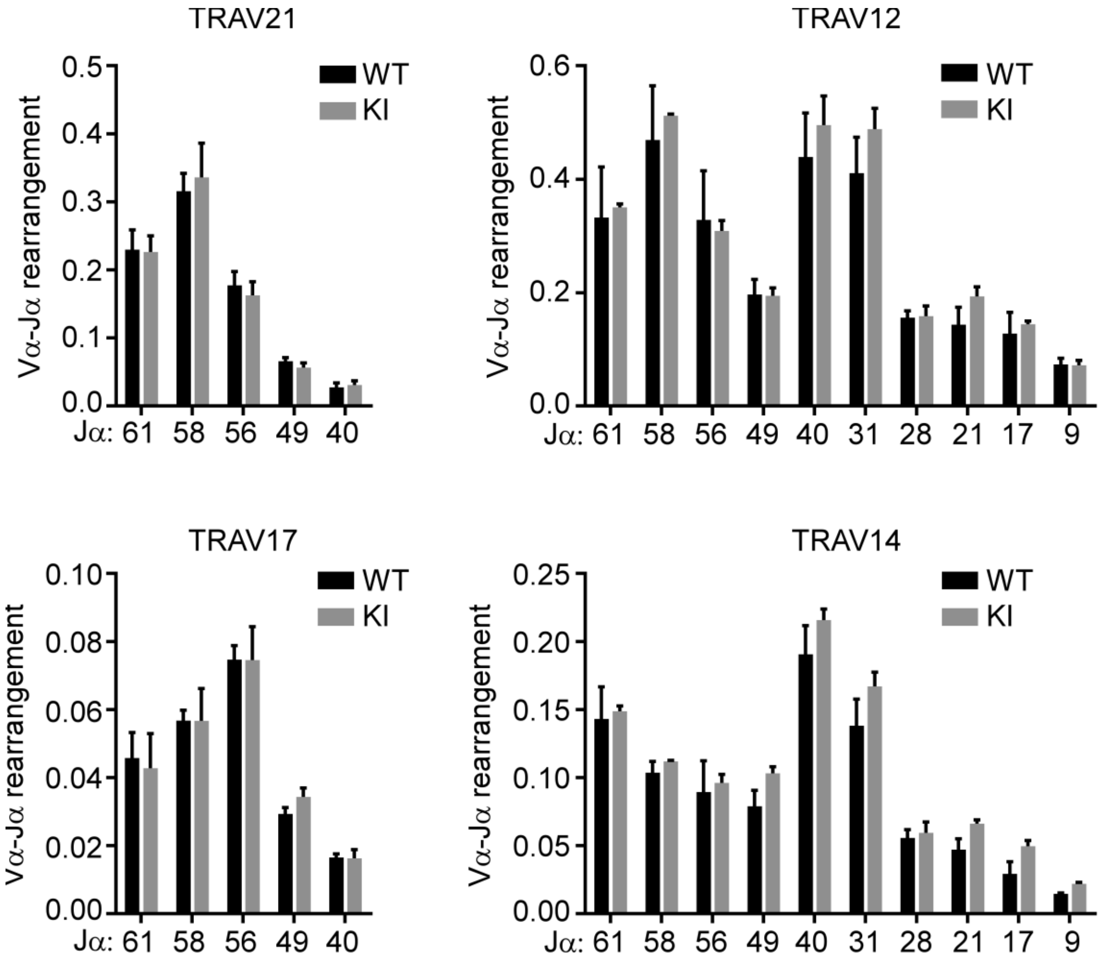

Supplemental Figure 5. Unperturbed Vα-Jα rearrangement in KI mice. Vα-Jα rearrangement was analyzed in genomic DNA isolated from purified DP thymocytes by real-time PCR. TRAV21 and TRAV17 primers detect individual V segments in the Jα-proximal portion of the Vα array which rearrange in early DP thymocytes to the most 5' Jα gene segments. TRAV12 and TRAV14 primers detect V segment families whose members are more Jα-distal and distributed across the Vα array. These V segments rearrange broadly across the Jα array in early and late DP thymocytes. Rearrangement data are presented as the mean±SEM of three DP thymocyte genomic DNA samples for each genotype.

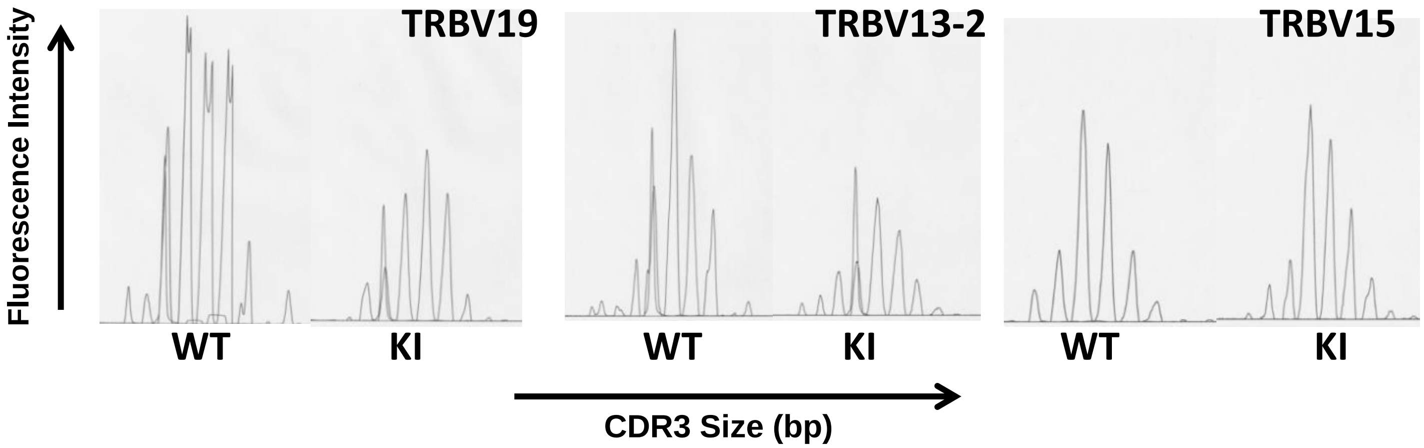

Supplementary Figure 6. Spectratype data from WT and KI CD4 cells showing reduced representation of TRBV19 (formerly Vβ6) and TRBV13-2 (formerly Vβ8.2) CD4 cells in the KI compared to WT mice while TRBV15 (formerly Vβ12) remained unchanged.

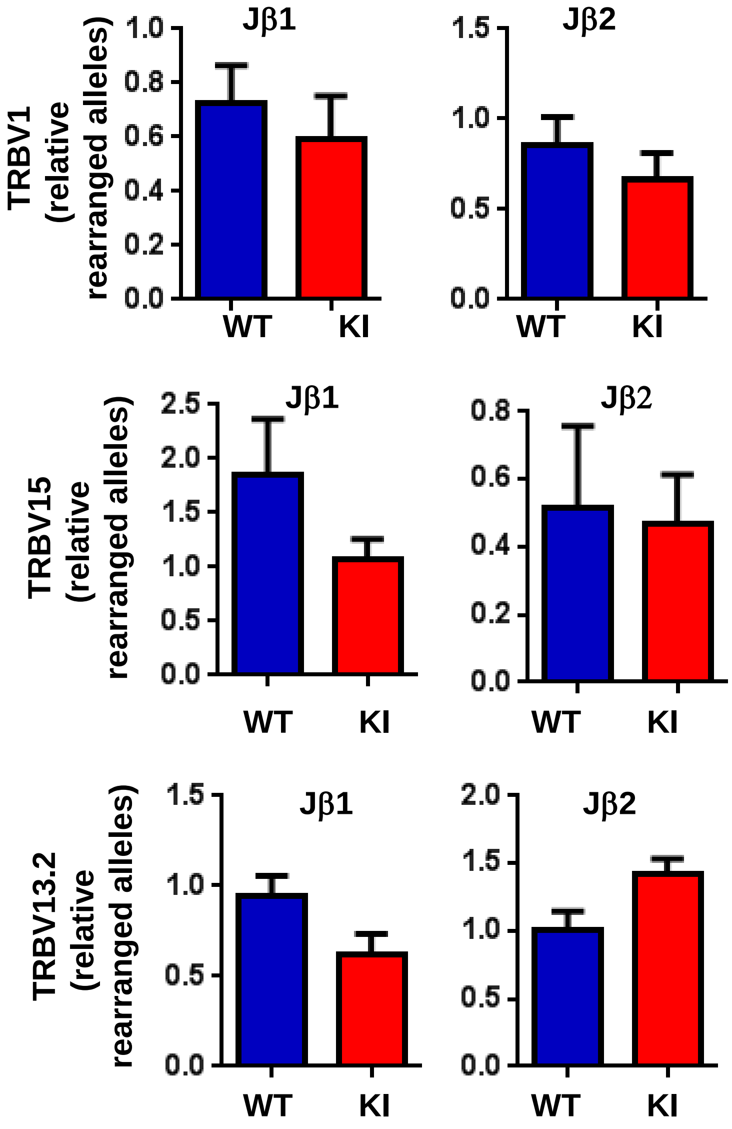

Supplementary Figure 7. Real-time PCR was used to examine recombination between Vβ and Jβ in genomic DNA from DN thymocytes. Signals were normalized to the non-recombining MAPK14 gene. Average levels from 3 independent DN preparations for each genotype are shown (n=3, mean±SEM). P >0.05 determined by t-test.

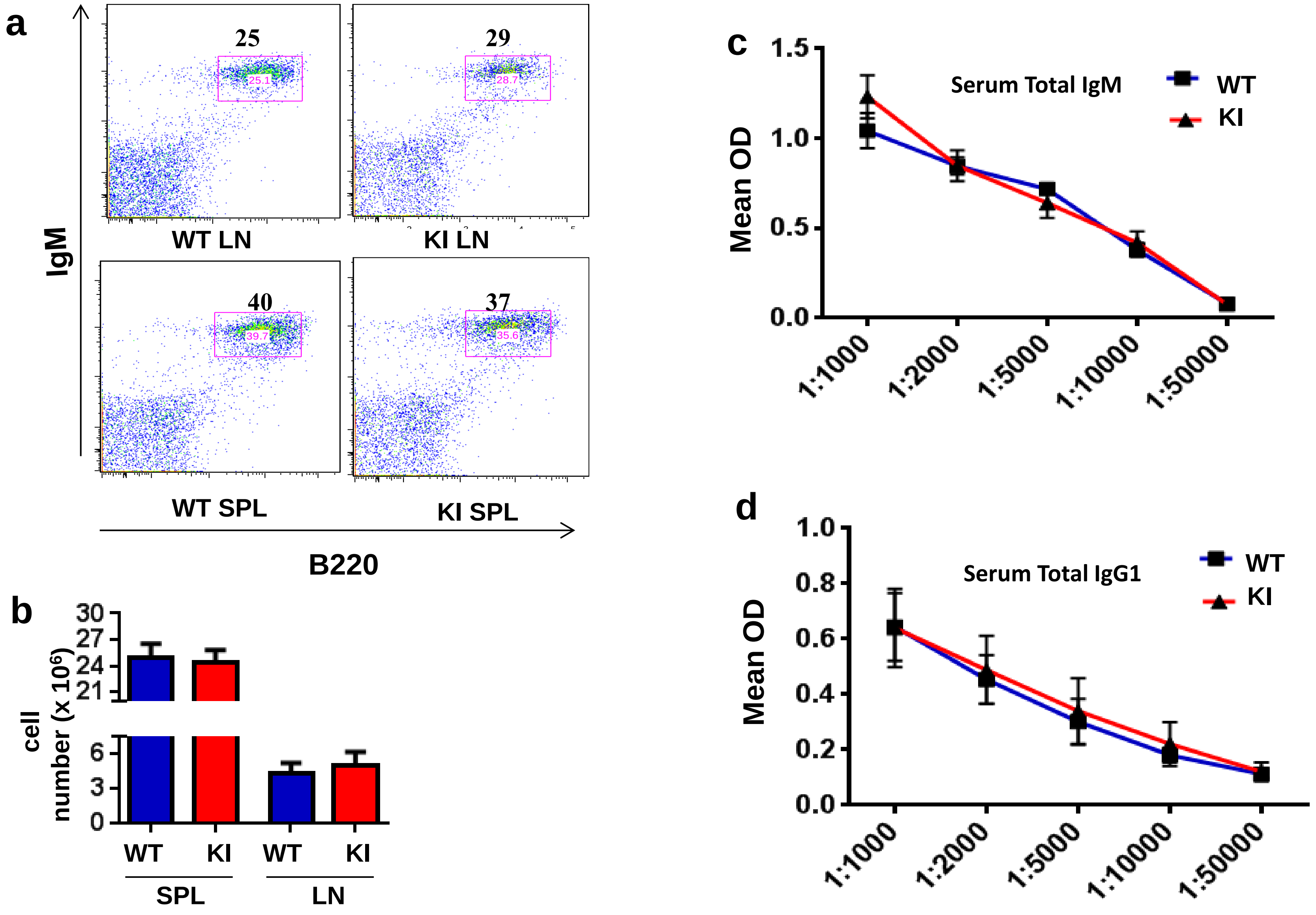

Supplementary Figure 8. (**a**) Representative FACS plots showing the percentages of B cells (IgM+ B220+) in the lymph nodes and spleens from WT and GSK3β-KI mice. (**b**) The number of B cells in spleen and lymph nodes from WT and GSK3β KI mice (n=3). (**c** and **d**) The levels of total IgM (**c**) and IgG1 (**d**) in the serum of WT and GSK3β KI mice were determined by ELISA.

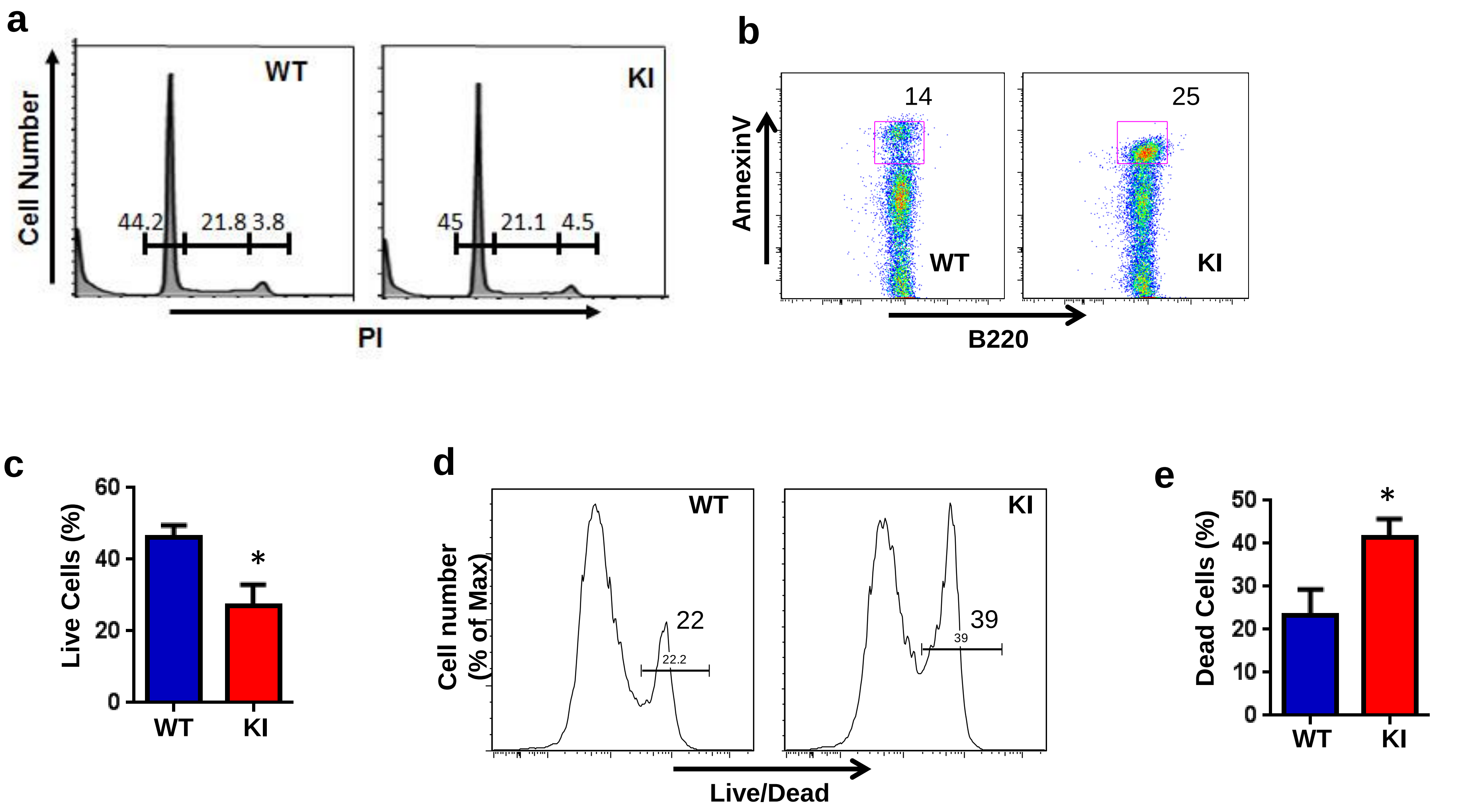

Supplementary Figure 9. (**a**) WT and GSK3β-KI B cells were activated for 48 hours and stained with PI to determine DNA content by flow cytometry analysis. Numbers represent the relative percentage of cells in the G1, S or the G2/M gates. (**b**) Representative flow cytometry dot plots showing Annexin V and B220 staining in activated (Day 3) B cells from WT and GSK3β-KI mice. Numbers represent the percentage of B220 cells positive for Annexin V. (**c**) The percentage of live WT and GSK3β-KI B cells (Day 3) based on side scatter (SSC) and forward scatter (FSC) examined by flow cytometry analysis (n=3). (**d**) Representative flow cytometry histogram showing Live/Dead staining in activated (Day 3)WT and GSK3β-KI B cells. Numbers show the percentage of dead cells. (**e**) The percentage of dead cells in WT and GSK3β-KI B cells after 3 days of activation(n=3). (*) indicates p value < 0.05 as determined by paired t- test.

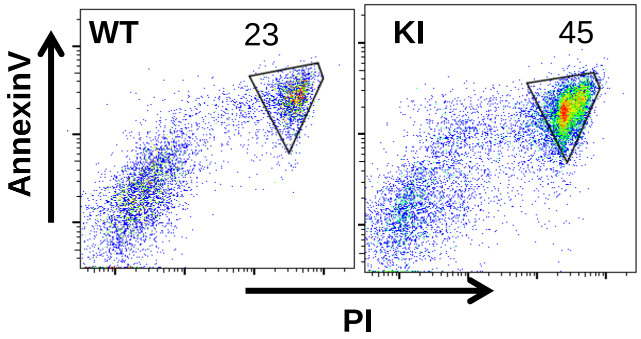

Supplementary Figure 10. WT and GSK3β-KI B cells were activated for 3 days, stained with AnnexinV and PI and analyzed by flow cytometry. Representative flow cytometry dot plots are shown. Numbers indicate the percentage of AnnexinV and PI double positive cells.

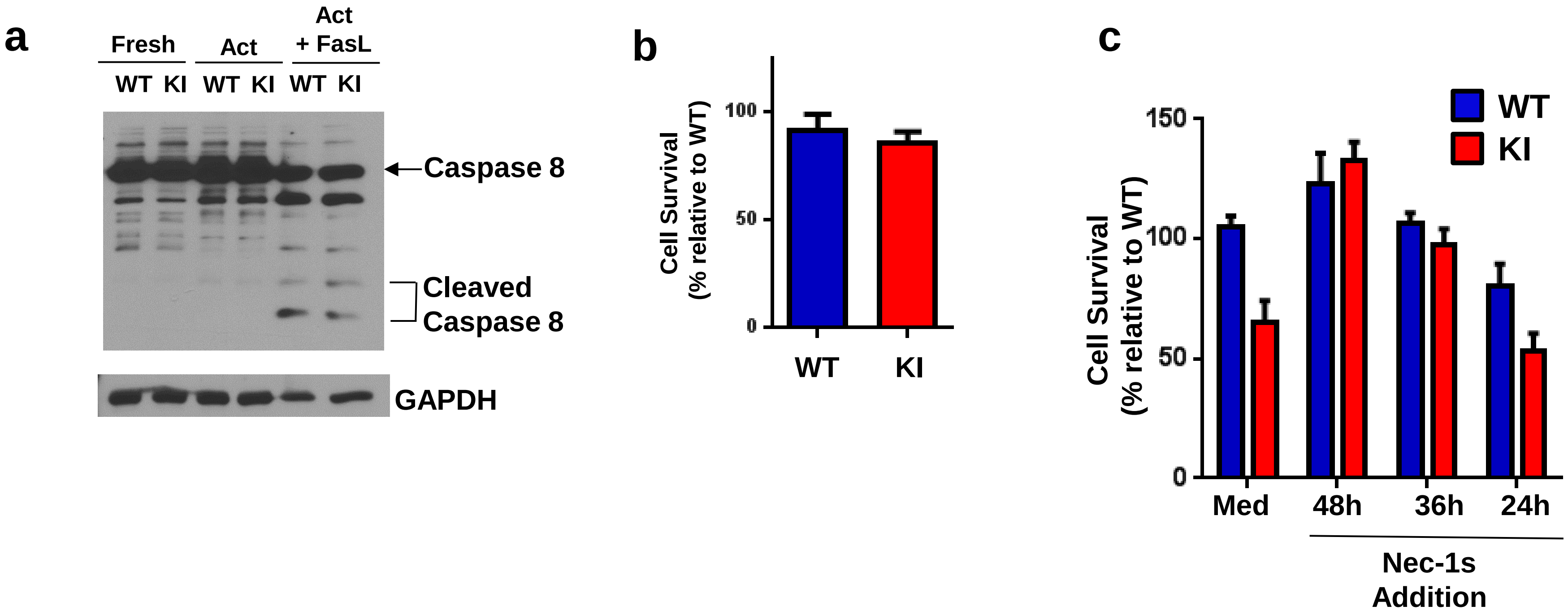

Supplementary Figure 11. (**a**) WT and GSK3β-KI B cells freshly isolated, activated for 2 days, or activated for two days and stimulated for 3 hours with FasL were examined for Caspase 8 by Western blot analysis. (**b**) B cells from WT and GSK3b-KI mice (n=3) were activated for 48 hours and viability determined by trypan blue staining. Percentage of cell survival relative to activated WT B cells is provided (% relative to WT cells). (**c**) WT and GSK3β-KI B cells were activated, Nectrostatin-1s (Nec-1s) was added 24h, 36h and 48h after activation and cell viability was determined by cell counting on day three of activation (n=3).

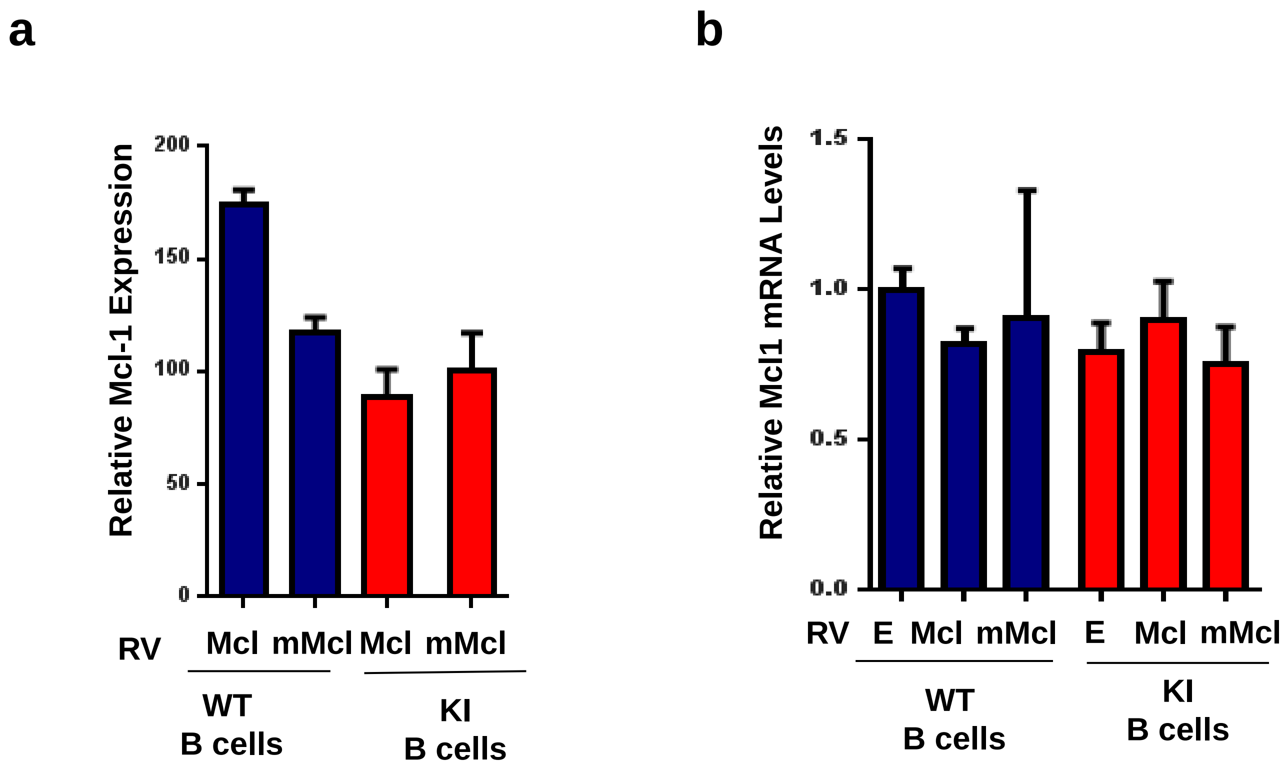

Supplementary Figure 12. WT and GSK3β-KI B cells were transduced with an empty retrovirus (E), a retrovirus expressing wildtype Mcl1 (Mcl) or a retrovirus expressing a Ser^{140}Ala mutant of Mcl1 (mMcl). (**a**) Three days after activation the transduction efficiency was examined by PCR for retrovirus Mcl1 in DNA isolated from transduced WT and GSK3β-KI B cells . Mcl-1 signal was normalized to the amount of input DNA. (**b**) Real time qRT-PCR was also used to detect the total levels of Mcl1 in WT and GSK3β-KI B cells. Expression of Mcl-1 relative to 18S rRNA is presented.

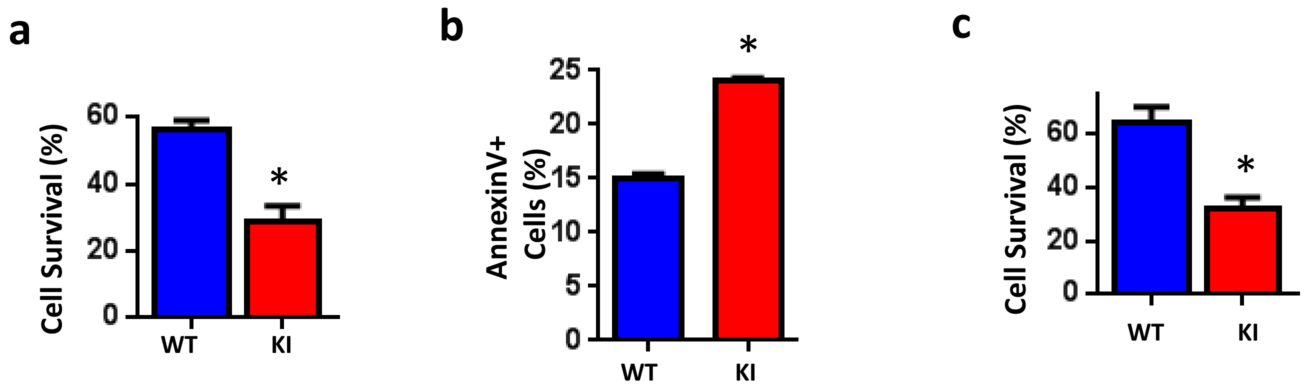

Supplementary Figure 13. (**a** and **b**) CD4 cells from WT and GSK3β–KI mice (n=4) were treated with Doxorubicin (Dox) and analyzed after 20h. (**a**) Cell survival was determined by trypan blue staining. The percentage of live cells relative to the initial cell number plated is shown. (**b**) Cell death was measured by AnnexinV staining and flow cytometry analysis. The percentage of AnnexinV positive cells in the recovered cells for each group is shown. (**c**) CD4 cells from WT and GSK3β–KI mice (n=3) were exposed to 5 Gy of X-rays and after 20h, cell survival was determined by trypan blue staining. The percentage of live cells relative to the initial cell number plated is shown. (*) indicates p value < 0.05 as determined by t- test.

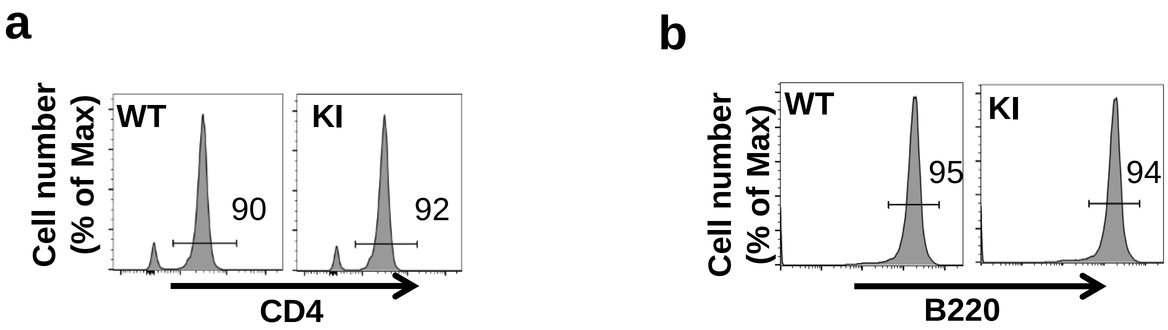

Supplementary Figure 14. (**a**) CD4 cells were purified from WT and GSK3β-KI mice. Representative histograms showing the percentage of CD4 positive cells in the purified cell populations. The number indicates the percentage of cells in the CD4 positive gate. (**b**) B cells were purified from WT and GSK3β-KI mice. Representative histograms showing the percentage of B220 positive cells in the purified cell populations. The number indicates the percentage of cells in the B220 positive gate.

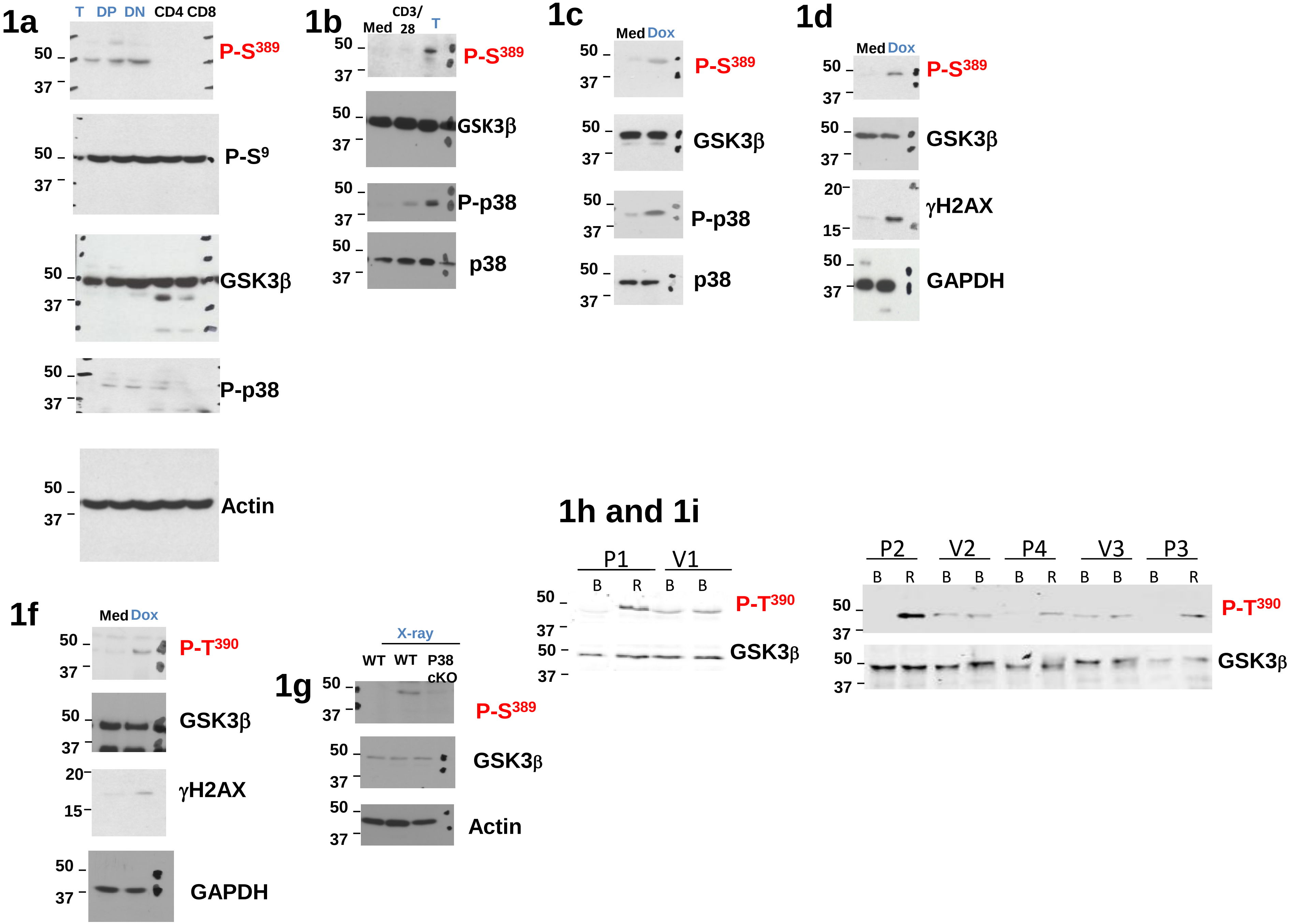

Supplementary Figure 15. Full scans the Western blots for the indicated figures.

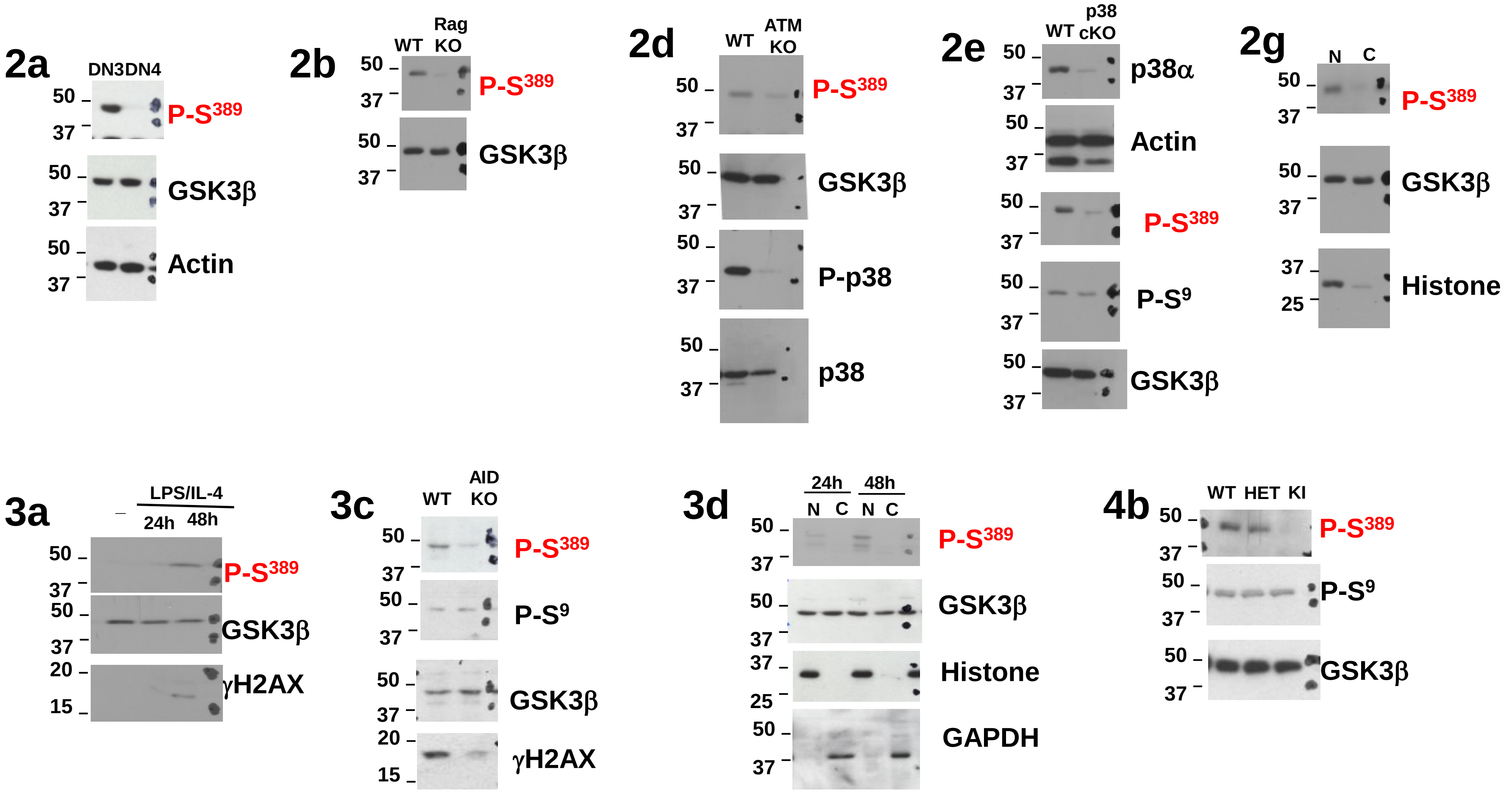

Supplementary Figure 16. Full scans the Western blots for the indicated figures.

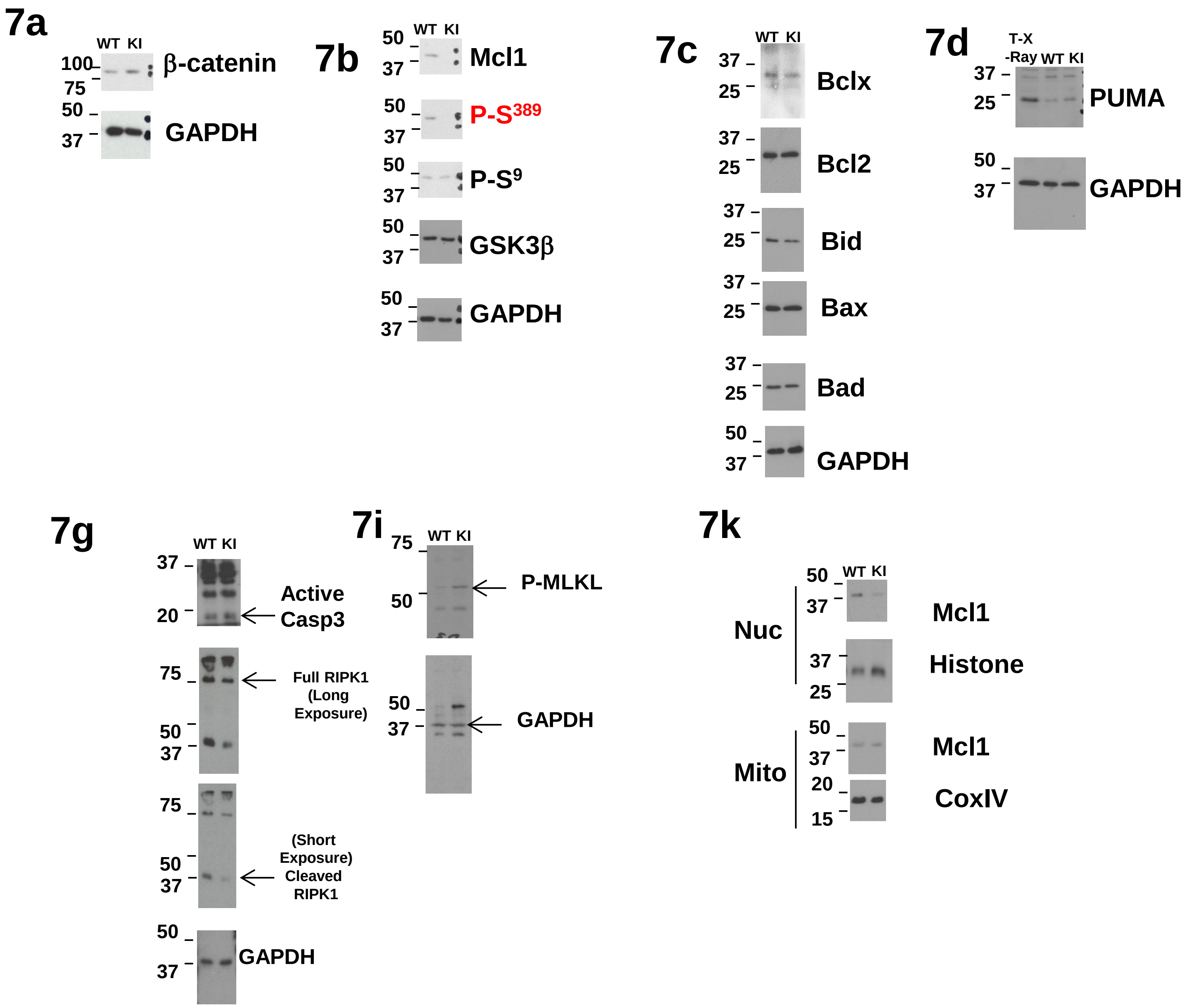

Supplementary Figure 17. Full scans the Western blots for the indicated figures.

SFig1 b

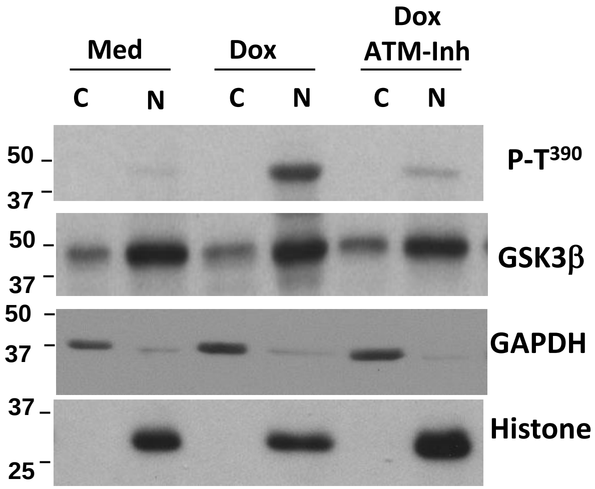

SFig11

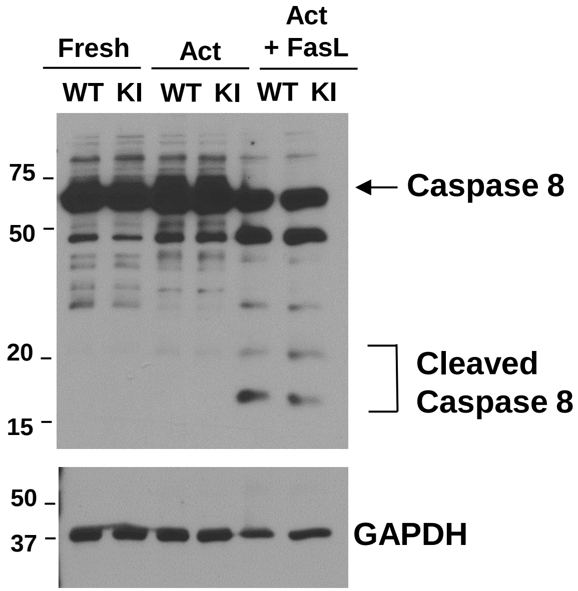

Supplementary Figure 18. Full scans the Western blots for the indicated figures.

Supplementary Table 1. Primers for TCRα Rearrangement

**TRAV12**	**5’-GCAGCAGCTCCTTCCATC-3’**
**TRAV14**	5’-TGGAGACTCAGCCACCTACT-3’
**TRAV17**	5’-TGGAGCGACTCAGCCAAGTA-3’
**TRAV21**	5’-GTATGGCTTTCCTGGCTATTGC-3’
**TRAJ61**	5’-ATGAGTCTTCCAGTCATGGC-3’
**TRAJ58**	5’-GACTCACTGTGAGCTTTGCC-3’
**TRAJ56**	5’-ACTCAGAACGGTTCCTTGACC-3’
**TRAJ49**	5’-GGAATGACAGTCAAACTTGTTCC-3’
**TRAJ40**	5’-TGGTACCTGCTCCAAAGACG-3’
**TRAJ31**	5’-GCGTCCCATCACCAAAGAAG-3’
**TRAJ28**	5’-GAGTGAGAATTTGGTGCCTTTC-3’
**TRAJ21**	5’-GGTGCCAGATCCGAAGTAAA-3’
**TRAJ17**	5’-TGATGGCTAGGCTCCTTTTC-3’
**TRAJ9**	5’-TGTCCCGAAGGTAAGTTTGTAG-3’
**Eα F**	5’-AGGAAGTCGCAGAACCTGAA-3’
**Eα R**	5’-GAGGGAGAAAGCCTTTTGGT-3’

Supplementary Table 2. Primers for TCRβ Rearrangement

TRBV1F	5'-GCCACACGGGTCACTGATAC-3'
TRBV13.2F	5'-GCTGGCAGCACTGAGAAAGGA-3'
TRBV15F	5'-CACTCTGAAGATTCAACCT-3'
TRBV16F	5'-CTCAGCTCAGATGCCCAAT-3'
TRBV19F	5'-CTCGAGAAGAAGTCATCT-3'
qJb1.1R	5'-CTCGAATATGGACACGGAGGACATGC-3'
qJb2.1R	5'-CCTGATACAGGGCCTTGGATAGTTA-3'
MAPK14 A3	5'-ATGAGATGCAGTACCCTTGGAGACCAGAAG-3'
MAPK14 A4	5'-AGCCAGGGCTATACAGAGAAAAACCCTGTG-3'
